# Synthesis, Structural and Antioxidant Studies of Some Novel *N*-Ethyl Phthalimide Esters

**DOI:** 10.1371/journal.pone.0119440

**Published:** 2015-03-05

**Authors:** C. S. Chidan Kumar, Wan-Sin Loh, Siddegowda Chandraju, Yip-Foo Win, Weng Kang Tan, Ching Kheng Quah, Hoong-Kun Fun

**Affiliations:** 1 X-ray Crystallography Unit, School of Physics, Universiti Sains Malaysia, Penang, Malaysia; 2 Department of Chemistry, Alva’s Institute of Engineering & Technology, Mijar, Karnataka, India; 3 Department of Sugar Technology & Chemistry, University of Mysore, Sir M.V. PG Center, Tubinakere, Karnataka, India; 4 Department of Chemical Science, Faculty of Science, Universiti Tunku Abdul Rahman, Perak Campus, Jalan Universiti, Bandar Barat, Perak, Malaysia; 5 Klinik Kesihatan Batu Kawa, Jalan Stapok Utara/ Utara, Kuching, Sarawak, Malaysia; 6 Department of Pharmaceutical Chemistry, College of Pharmacy, King Saud University, Riyadh, Saudi Arabia; The University of Iowa, UNITED STATES

## Abstract

A series of *N*-ethyl phthalimide esters **4(a-n)** were synthesized and characterized by spectroscopic studies. Further, the molecular structure of majority of compounds were analysed by single crystal X-ray diffraction studies. The X-ray analysis revealed the importance of substituents on the crystal stability and molecular packing. All the synthesized compounds were tested for *in vitro* antioxidant activity by DPPH radical scavenging, FRAP and CUPRAC methods. Few of them have shown good antioxidant activity.

## Introduction

The chemistry of heterocyclic compounds is one of the most complex branches of organic chemistry. Heterocyclic compounds play vital role in biological processes and the researchers are trying to understand the chemistry of heterocyclic compounds in order to improve the quality of daily life [1]. Major fractions of organic compounds isolated from nature are comprised of nitrogen heterocycles. Numerous lines of evidence suggest that heterocyclic compounds used as analgesic, anti-inflammatory and anti-migraine agents can be potent regulators of the nitroxidative stress and targeting free nitrogen and oxygen radicals is a very promising strategy for future pain management [2]. The structural diversity and biological importance of nitrogen containing heterocycles have made them attractive targets for synthesis over many years. Constructing highly functionalized heterocyclic compounds would seem to be essential and significant. The 5-membered *N*-heterocycles are of exceptional interest in the pharmaceutical industry, as they appear in the core structure of several drugs. Among heterocyclic scaffolds, phthalimides are of particular biological interest and have been reported as herbicides, insecticides, antipsychotics and anti-inflammatory agents. Generally, in organic synthesis, they are used as starting materials and intermediates for the synthesis of variety of bioactive compounds. The use of phthalimides as primary amine protecting groups is extensively documented in the chemical literature, especially for α–amino acids. Substituted phthalimides are used predominantly as chiral building blocks in organic synthesis and can be used as key intermediates in the preparation of bio-active compounds i.e. antibacterial, analgesic, antifungal, virucidal, plant growth regulator and also in dye industry. In view of their significant roles in biological activities, such as anti-inflamatary [3], hypolipidemic [4], analgesic [5] and other biomedical activities [6], therefore, development of new and efficient methodologies for these bioactive compounds is important. Research accounts in the field of organic chemistry and synthesis of five-membered nitrogen heterocyclic compounds (both aromatic and non-aromatic) as well as natural products with such heterocyclic systems, is still an open challenge.

Encouraged with the above findings, herein we are reporting the synthesis, structural and antioxidant studies of several novel phthalimide ester derivatives by introducing ester group to the main core structure. In the present study, the spacer of one carbon atom distance was introduced to connect the *N*-terminius of the phthalimide with ester group and to investigate their antioxidant properties.

## Materials and Methods

The reagents and solvents for the synthesis were obtained from the Aldrich Chemical Co., and were used without additional purification. The purity of each compound was confirmed by thin layer chromatography using Merck silica gel 60 F254-coated aluminium plates. Open capillary method was employed to determine the melting points and were found uncorrected.

The infrared spectra were recorded using a Perkin-Elmer System 2000 FTIR Spectrophotometer as KBr disc from 4000–400 cm^-1^. The spectra for ^1^H, ^13^C and ^1^H-^13^C HMQC NMR were recorded on a JEOL JNM-ECX 400 FT-NMR Spectrometer using deuterated CDCl_3_ as the solvent and tetramethylsilane, TMS as the internal standard. Elemental analyses (CHN) were carried out on a Perkin Elmer Series II, 2400 analyzer. X-ray analysis was done using Apex II Duo CCDC diffractometer. The data were processed with SAINT and absorption correction was done using SADABS [7]. The structures were solved by direct method using the program SHELXTL [8], and were refined by full-matrix lowest squares technique on *F* ^*2*^ using anisotropic displacement parameters. The non-hydrogen atoms were refined anisotropically. In these compounds, all the H atoms were calculated geometrically with isotropic displacement parameters set to 1.2 (1.5 for methyl groups) times the equivalent isotropic *U* values of the parent carbon atoms. The overlay structures were drawn using Olex2 software [9]. Crystallographic data for compounds **4(a-n)** (excluding **4d**, **4h** and **4m**) have been deposited at the Cambridge Crystallographic Data Centre. S1 Supporting Information contains the checkCIF report of these deposited compounds. Copies of the data can be obtained free of charge on application to the CCDC, 12 Union Road, Cambridge CB2 IEZ, UK. Fax: +44-(0)1223–336033 or E-Mail: deposit@ccdc.cam.ac.uk.

### Synthesis

**Fig 1:**
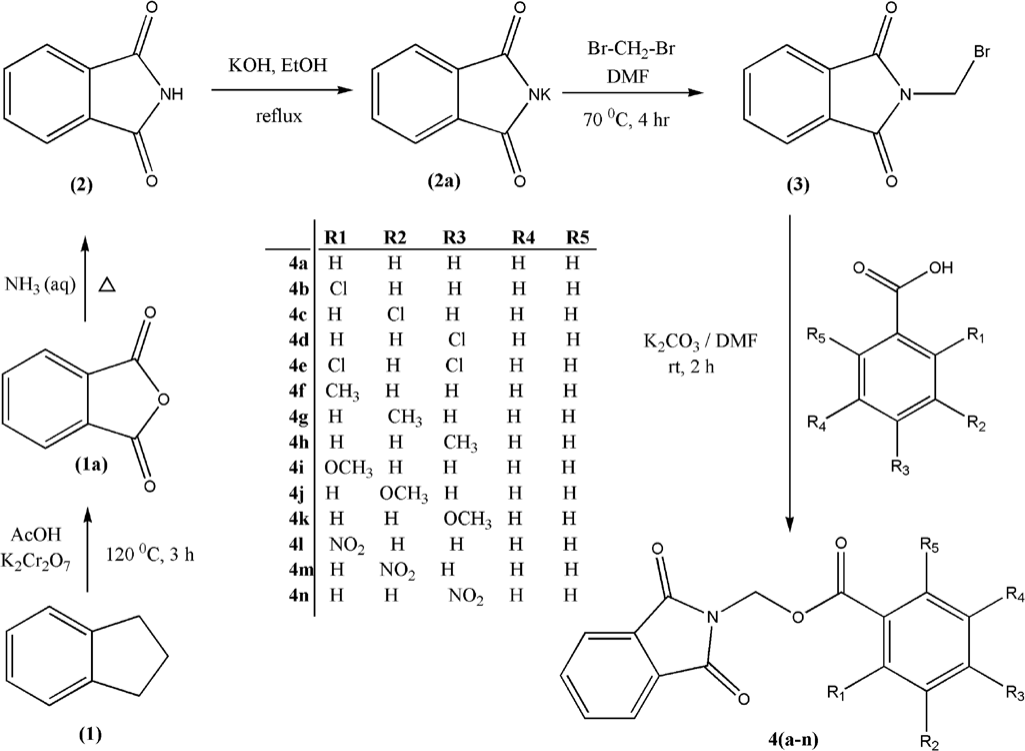
Synthesis of (1,3‐dioxo‐2,3‐dihydro‐1*H*‐isoindol‐2‐yl)methyl (substituted) benzoates **4(a-n)**.

The systematic procedure followed for the synthesis of target compounds is depicted in Fig. 1. 20.0 g (0.085 mol) of indane (**1**) was taken in 500 mL round-bottomed flask, and then added with 200 mL of acetic acid and potassium dichromate (76.7 g, 0.257 mol). The reaction mixture was refluxed for 3 h at 120°C. The solvent was removed in vacuum, the chromium salt was removed from the residue using boiling water and the white crude product was recrystallized from glacial acetic acid to give white needle precipitates (**1a**). In a 250 mL round bottom flask, powdered phthalic anhydride (**1a**; 15 g) was mixed with aqueous ammonia (11 mL) and fitted with a wide air condenser. The reaction mixture was heated at reflux temperature until a homogeneous melt is formed. All the water gets evaporated during the first hour of the reaction time. The flask was shaken occasionally, the heating was continued and any material which sublimes into condenser was push down with a glass rod. After the completion of reaction, the contents were allowed to cool, dried and recrystallized to get a fine powder of **2** (95%).

The synthesized compound (**2**) (10.0 g, 0.036 mol) was dispersed in a solution of potassium hydroxide in ethanol (100 mL). The solution was refluxed for 4 h; the liquor was then cooled and the product (**2a**) was filtered, washed successively with water and dried in vacuum. 0.3 mol of potassium phthalimide (**2a**) was reacted with dibromomethane (10 mol) in 30 mL of DMF. The reaction mixture was stirred for 4 h at 70°C. The liquor was then cooled and the product was filtered, washed with water and dried in vacuum. The precipitate was recrystallized in ethanol to obtain the pure compound (**3**).

#### Synthesis of (1,3‐dioxo‐2,3‐dihydro‐1H‐isoindol‐2‐yl)methyl (substituted)benzoates 4(a-n)

The vacuum dried intermediate **3** (0.002 mol) was then reacted with mono- and di-substituted benzoic acids (0.003 mol) separately in presence of anhydrous potassium carbonate (0.003 mol) in DMF (10 mL). The reaction mixture was stirred at room temperature for about 2 h. The progress of the reaction was monitored by TLC. After completion of the reaction, the reaction mixture was poured into ice-cold water and allowed stir for 10 min. The solid obtained was filtered and washed successively with distilled water and recrystallized from ethanol to obtain **4(a–n)**.

#### (1,3-Dioxo-2,3-dihydro-1H-isoindol-2-yl)methyl benzoate (4a)

Isolated yield 78%; Solvent for growing crystals: Acetone; M. P.: 149–151°C; FTIR as KBr disc (cm^-1^): ν (C-H) aromatic 3062, 3049, 3024; ν(C = O) 1782, 1726; ν(C = C) 1448, ν(C-N) 1298, ν(C-O) 1279. ^1^H-NMR (ppm) (CDCl_3_): δ: aromatic protons 7.40 (t, 5.9 Hz, 2H); 7.54 (t, 7.3 Hz, 1H); 7.79 (q, 2.3 Hz, 2H); 7.94 (q, 2.8 Hz, 2H); 8.01 (d, 6.9 Hz, 2H); CH_2_ 5.97 (s, 2H). ^13^C-NMR (ppm) (CDCl_3_): δ: aromatic carbons 124.10, 128.49, 129.19, 129.98, 131.86, 133.53, 134.78; CH_2_ 61.36, COO 165.50, C = O 166.86. Anal. Calc. for C_16_H_11_NO_4_: C, 68.32; H, 3.94; N, 4.98. Found: C, 68.29; H, 3.97; N, 4.99. CCDC No.: 1019340.

#### (1,3-Dioxo-2,3-dihydro-1H-isoindol-2-yl)methyl 2-chlorobenzoate (4b)

Isolated yield 74%; Solvent for growing crystals: Mixture of acetone and ethanol (1:1 v/v); M. P.: 161–163°C; FTIR as KBr disc (cm^-1^): ν(C-H) aromatic 3099, 3043; ν(C-H) aliphatic 2970, ν(C = O) 1782, 1735; ν(C = C) 1591, 1470; ν(C-N) 1298, ν(C-O) 1244, ν(C-Cl) 1038. ^1^H-NMR (ppm) (CDCl_3_): δ: aromatic protons 7.27 (t, 5.9 Hz, 1H); 7.41 (q, 6.4 Hz, 2H); 7.78 (q, 2.3 Hz, 2H); 7.82 (s, 1H); 7.94 (q, 2.3 Hz, 2H); CH_2_ 5.96 (s, 2H). ^13^C-NMR (ppm) (CDCl_3_): δ: aromatic carbons 124.14, 126.65, 128.86, 131.29, 131.83, 131.88, 133.16, 134.30 134.81; CH_2_ 61.75, COO 164.38, C = O 166.76. Anal. Calc. for C_16_H_10_ClNO_4_: C, 60.87; H, 3.19; N, 4.44. Found: C, 60.85; H, 3.17; N, 4.43. CCDC No.: 1019275.

#### (1,3-Dioxo-2,3-dihydro-1H-isoindol-2-yl)methyl 3-chlorobenzoate (4c)

Isolated yield 81%; Solvent for growing crystals: Mixture of acetone and ethanol (1:1 v/v); M. P.: 157–159°C; FTIR as KBr disc (cm^-1^): ν(C-H) aromatic 3103, 3091, 3073, 3047, 3033; ν(C-H) aliphatic 2975, ν(C = O) 1782, 1728; ν(C = C) 1597, 1470; ν(C-N) 1312, ν(C-O) 1251, ν(C-Cl) 1071. ^1^H-NMR (ppm) (CDCl_3_): δ: aromatic protons 7.35 (t, 7.8 Hz, 1H); 7.51 (d, 8.3 Hz, 1H); 7.79 (q, 2.3 Hz, 2H); 7.89 (d, 7.8 Hz, 1H); 7.94 (q, 2.3 Hz, 2H); 7.97 (t, 1.8 Hz, 1H); CH_2_ 5.96 (s, 2H). ^13^C-NMR (ppm) (CDCl_3_): δ: aromatic carbons 124.16, 128.12, 129.84, 129.95, 130.92, 131.82, 133.57, 134.66 134.85; CH_2_ 61.59, COO 164.32, C = O 166.78. Anal. Calc. for C_16_H_10_ClNO_4_: C, 60.87; H, 3.19; N, 4.44. Found: 60.84; H, 3.32; N, 4.42. CCDC No.: 1019341.

#### (1,3-Dioxo-2,3-dihydro-1H-isoindol-2-yl)methyl 4-chlorobenzoate (4d)

Isolated yield 79%; Solvent for growing crystals: Mixture of acetone and methanol (1:1 v/v); M. P.: 145–147°C; FTIR as KBr disc (cm^-1^): ν(C-H) aromatic 3101, 3072, 3044; ν(C-H) aliphatic 2968, ν(C = O) 1785, 1735; ν(C = C) 1594, ν(C-N) 1306, ν(C-O) 1270, ν(C-Cl) 1095. ^1^H-NMR (ppm) (CDCl_3_): δ: aromatic protons 7.38 (d, 8.7 Hz, 2H); 7.79 (q, 2.3 Hz, 2H); 7.94 (q, 2.3 Hz, 4H); CH_2_ 5.95 (s, 2H). ^13^C-NMR (ppm) (CDCl_3_): δ: aromatic carbons 124.14, 127.64, 128.87, 131.36, 131.82, 134.83, 140.05; CH_2_ 61.47, COO 164.65, C = O 166.81. Anal. Calc. for C_16_H_10_ClNO_4_: C, 60.87; H, 3.19; N, 4.44. Found: C, 60.84; H, 3.21; N, 4.41.

#### (1,3-Dioxo-2,3-dihydro-1H-isoindol-2-yl)methyl 2,4-dichlorobenzoate (4e)

Isolated yield 76%; Solvent for growing crystals: Acetone; M. P.: 134–136°C; FTIR as KBr disc (cm^-1^): ν(C-H) aromatic 3093, 3067, 3046; ν(C-H) aliphatic 2977, ν(C = O) 1783, 1727; ν(C = C) 1586, 1470; ν(C-N) 1311, ν(C-O) 1277, ν(C-Cl) 1046. ^1^H-NMR (ppm) (CDCl_3_): δ: aromatic protons 7.25 (qn, 1.8 Hz, 1H); 7.44 (d, 1.8 Hz, 1H); 7.78–7.82 (m, 3H); 7.94 (q, 2.3 Hz, 2H); CH_2_ 5.95 (s, 2H). ^13^C-NMR (ppm) (CDCl_3_): δ: aromatic carbons 124.17, 127.10, 131.25, 131.79, 132.96, 134.87, 135.54, 139.04; CH_2_ 61.83, COO 163.46, C = O 166.71. Anal. Calc. for C_16_H_9_Cl_2_NO_4_: C, 54.88; H, 2.59; N, 4.00. Found: C, 54.85; H, 2.61; N, 3.98. CCDC No.: 1019342.

#### (1,3-Dioxo-2,3-dihydro-1H-isoindol-2-yl)methyl 2-methylbenzoate (4f)

Isolated yield 80%; Solvent for growing crystals: Mixture of acetone and methanol (1:1 v/v); M. P.: 144–146°C; FTIR as KBr disc (cm^-1^): ν(C-H) aromatic 3101, 3045, 3029; ν(C-H) aliphatic 2971, 2924; ν(C = O) 1783, 1732; ν(C = C) 1602, 1459; ν(C-N) 1315, ν(C-O) 1241. ^1^H-NMR (ppm) (CDCl_3_): δ: aromatic protons 7.19 (q, 7.7 Hz, 2H); 7.38 (t, 7.3 Hz, 1H); 7.78 (q, 2.3 Hz, 2H); 7.87 (d, 7.8 Hz, 1H); 7.94 (q, 2.7 Hz, 2H); CH_3_ 2.58 (s, 3H); CH_2_ 5.94 (s, 2H). ^13^C-NMR (ppm) (CDCl_3_): δ: aromatic carbons 124.07, 125.79, 128.44, 131.00, 131.82, 131.87, 132.57, 134.73, 140.86; CH_3_ 21.84, CH_2_ 61.23, COO 166.25, C = O 166.88. Anal. Calc. for C_17_H_13_NO_4_: C, 69.15; H, 4.44; N, 4.74. Found: C, 69.12; H, 4.46; N, 4.71. CCDC No.: 1019276.

#### (1,3-Dioxo-2,3-dihydro-1H-isoindol-2-yl)methyl 3-methylbenzoate (4g)

Isolated yield 74%; Solvent for growing crystals: Acetone; M. P.: 148–150°C; FTIR as KBr disc (cm^-1^): ν(C-H) aromatic 3060, ν(C-H) aliphatic 2965, 2923; ν(C = O) 1777, 1725; ν(C = C) 1610, 1459; ν(C-N) 1311, ν(C-O) 1275. ^1^H-NMR (ppm) (CDCl_3_): δ: aromatic protons 7.28 (t, 7.8 Hz, 1H); 7.34 (d, 7.4 Hz, 1H); 7.78 (q, 2.3 Hz, 2H); 7.82 (s, 2H); 7.94 (q, 2.7 Hz, 2H); CH_3_ 2.34 (s, 3H); CH_2_ 5.96 (s, 2H). ^13^C-NMR (ppm) (CDCl_3_): δ: aromatic carbons 124.08, 127.15, 128.37, 129.11, 130.44, 131.89, 134.29, 134.74, 138.30; CH_3_ 21.29, CH_2_ 61.33, COO 165.69, C = O 166.87. Anal. Calc. for C_17_H_13_NO_4_: C, 69.15; H, 4.44; N, 4.74. Found: C, 69.13; H, 4.46; N, 4.72. CCDC No.: 1019277.

#### (1,3-Dioxo-2,3-dihydro-1H-isoindol-2-yl)methyl 4-methylbenzoate (4h)

Isolated yield 80%; Solvent for growing crystals: Mixture of acetone and methanol (1:1 v/v); M. P.: 139–141°C; FTIR as KBr disc (cm^-1^): ν(C-H) aromatic 3101, 3031; ν(C-H) aliphatic 2974, 2923; ν(C = O) 1782, 1727; ν(C = C) 1611, 1448; ν(C-N) 1307, ν(C-O) 1272. ^1^H-NMR (ppm) (CDCl_3_): δ: aromatic protons 7.19 (d, 7.8 Hz, 2H); 7.78 (q 2.3 Hz, 2H); 7.89 (d, 8.3 Hz, 2H); 7.93 (q, 2.3 Hz, 2H); CH_3_ 2.37 (s, 3H); CH_2_ 5.95 (s, 2H). ^13^C-NMR (ppm) (CDCl_3_): δ: aromatic carbons 124.07, 126.45, 129.19, 130.01, 131.89, 134.72, 144.30; CH_3_ 21.78, CH_2_ 61.23, COO 165.56, C = O 166.89. Anal. Calc. for C_17_H_13_NO_4_: C, 69.15; H, 4.44; N, 4.74. Found: C, 69.11; H, 4.45; N, 4.73.

#### (1,3-Dioxo-2,3-dihydro-1H-isoindol-2-yl)methyl 2-methoxybenzoate (4i)

Isolated yield 71%; Solvent for growing crystals: Mixture of acetone and methanol (1:1 v/v); M. P.: 144–146°C; FTIR as KBr disc (cm^-1^): ν(C-H) aromatic 3072, 3026; ν(C-H) aliphatic 2981, 2949, 2844; ν(C = O) 1786, 1742, 1721; ν(C = C) 1598, 1492; ν(C-N) 1331, ν(C-O) 1237. ^1^H-NMR (ppm) (CDCl_3_): δ: aromatic protons 6.89 (s, 1H); 6.93 (t, 8.2 Hz, 1H); 7.77 (q, 2.3 Hz, 2H); 7.44 (t, 6.9 Hz, 1H); 7.75 (d, 2.8 Hz, 1H); 7.93 (q, 2.3 Hz, 2H); OCH_3_ 3.86 (s, 3H); CH_2_ 5.92 (s, 2H). ^13^C-NMR (ppm) (CDCl_3_): δ: aromatic carbons 112.12, 118.81, 120.11, 124.02, 131.93, 132.03, 134.21, 134.68, 159.72; OCH_3_ 56.06, CH_2_ 61.39, COO 164.71, C = O 166.89. Anal. Calc. for C_17_H_13_NO_5_: C, 65.59; H, 4.21; N, 4.50. Found: C, 65.57; H, 4.24; N, 4.49. CCDC No.: 1019343.

#### (1,3-Dioxo-2,3-dihydro-1H-isoindol-2-yl)methyl 3-methoxybenzoate (4j)

Isolated yield 78%; Solvent for growing crystals: Mixture of acetone and methanol (1:1 v/v); M. P.: 133–135°C; FTIR as KBr disc (cm^-1^): ν(C-H) aromatic 3084, 3028; ν(C-H) aliphatic 2977, 2945, 2840; ν(C = O) 1780, 1736; ν(C = C) 1602, 1465; ν(C-N) 1323, ν(C-O) 1273. ^1^H-NMR (ppm) (CDCl_3_): δ: aromatic protons 7.08 (dd, 2.8 Hz, 0.92Hz, 1H); 7.29 (t, 7.8 Hz, 1H); 7.51 (s, 1H); 7.59 (d, 7.8 Hz, 1H); 7.78 (q, 2.3 Hz, 2H); 7.93 (q, 2.3 Hz, 2H); OCH_3_ 3.80 (s, 3H); CH_2_ 5.95 (s, 2H). ^13^C-NMR (ppm) (CDCl_3_): δ: aromatic carbons 114.28, 120.11, 122.40, 124.09, 129.52, 130.45, 131.85, 134.77, 159.60; OCH_3_ 55.56, CH_2_ 61.40, COO 165.39, C = O 166.84. Anal. Calc. for C_17_H_13_NO_5_: C, 65.59; H, 4.21; N, 4.50. Found: C, 65.57; H, 4.24; N, 4.48. CCDC No.: 1019278.

#### (1,3-Dioxo-2,3-dihydro-1H-isoindol-2-yl)methyl 4-methoxybenzoate (4k)

Isolated yield 82%; Solvent for growing crystals: Acetone; M. P.: 130–133°C; FTIR as KBr disc (cm^-1^): ν(C-H) aromatic 3097, 3046; ν(C-H) aliphatic 2964, 2945, 2845; ν(C = O) 1782, 1728; ν(C = C) 1607, 1471; ν(C-N) 1305, ν(C-O) 1258. ^1^H-NMR (ppm) (CDCl_3_): δ: aromatic protons 6.86 (d, 8.7 Hz, 2H); 7.77 (q, 2.3 Hz, 2H); 7.93 (q, 2.3 Hz, 2H); 7.96 (d, 11.0 Hz, 2H); OCH_3_ 3.86 (s, 3H); CH_2_ 5.93 (s, 2H). ^13^C-NMR (ppm) (CDCl_3_): δ: aromatic carbons 113.73, 121.55, 124.07, 131.89, 132.09, 134.72, 163.83; OCH_3_ 55.54, CH_2_ 61.13, COO 165.20, C = O 166.90. Anal. Calc. for C_17_H_13_NO_5_: C, 65.59; H, 4.21; N, 4.50. Found: C, 65.56; H, 4.23; N, 4.47. CCDC No.: 1019279.

#### (1,3-Dioxo-2,3-dihydro-1H-isoindol-2-yl)methyl 2-nitrobenzoate (4l)

Isolated yield 79%; Solvent for growing crystals: Mixture of acetone and methanol (1:1 v/v); M. P.: 152–154°C; FTIR as KBr disc (cm^-1^): ν(C-H) aromatic 3110, 3099, 3045; ν(C-H) aliphatic 2974, ν(C = O) 1784, 1733; ν(C = C) 1603, 1472; ν(NO_2_)1540, ν(C-N) 1315, ν(C-O) 1273. ^1^H-NMR (ppm) (CDCl_3_): δ: aromatic protons 7.59–7.68 (m, 2H); 7.73 (d, 5.6 Hz, 1.8 Hz, 1H); 7.78 (q, 2.3 Hz, 2H); 7.90 (d, 1.4 Hz, 1H); 7.94 (q, 2.3 Hz, 2H); CH_2_ 5.97 (s, 2H). ^13^C-NMR (ppm) (CDCl_3_): δ: aromatic carbons 124.14, 126.93, 130.01, 131.76, 132.13, 133.16, 134.48, 134.82, 148.00; CH_2_ 61.83, COO 164.35, C = O 166.57. Anal. Calc. for C_16_H_10_N_2_O_6_: C, 58.90; H, 3.09; N, 8.59. Found: C, 58.97; H, 3.12; N, 8.57. CCDC No.: 1019280.

#### (1,3-Dioxo-2,3-dihydro-1H-isoindol-2-yl)methyl 3-nitrobenzoate (4m)

Isolated yield 73%; Solvent for growing crystals: Mixture of acetone and methanol (1:1 v/v); M. P.: 157–159°C; FTIR as KBr disc (cm^-1^): ν(C-H) aromatic 3103, 3083; ν(C-H) aliphatic 2974, ν(C = O) 1785, 1725; ν(C = C) 1616, 1468; ν(NO_2_)1529, ν(C-N) 1314, ν(C-O) 1259. ^1^H-NMR (ppm) (CDCl_3_): δ: aromatic protons 7.64 (t, 8.2 Hz, 1H); 7.81 (q, 2.3 Hz, 2H); 7.96 (q, 2.3 Hz, 2H); 8.35 (d, 7.8 Hz, 1H); 8.41 (d, 8.2 Hz, 1H); 8.81 (s, 1H); CH_2_ 6.01 (s, 2H). ^13^C-NMR (ppm) (CDCl_3_): δ: aromatic carbons 124.25, 124.91, 127.97, 129.82, 130.99, 131.79, 134.94, 135.65, 148.34; CH_2_ 61.99, COO 163.49, C = O 166.73. Anal. Calc. for C_16_H_10_N_2_O_6_: C, 58.90; H, 3.09; N, 8.59. Found: C, 58.97; H, 3.11; N, 8.55.

#### (1,3-Dioxo-2,3-dihydro-1H-isoindol-2-yl)methyl 4-nitrobenzoate (4n)

Isolated yield 83%; Solvent for growing crystals: Mixture of acetone and methanol (1:1 v/v); M. P.: 161–163°C; FTIR as KBr disc (cm^-1^): ν(C-H) aromatic 3113, 3083, 3055; ν(C = O) 1784, 1740; ν(C = C) 1606, 1467; ν(NO_2_)1527, ν(C-N) 1324, ν(C-O) 1276. ^1^H-NMR (ppm) (CDCl_3_): δ: aromatic protons 7.81 (q, 2.3 Hz, 2H); 7.95 (q, 2.3 Hz, 2H); 8.18 (d, 8.7 Hz, 2H); 8.25 (d, 8.7 Hz, 2H); CH_2_ 6.00 (s, 2H). ^13^C-NMR (ppm) (CDCl_3_): δ: aromatic carbons 123.66, 124.24, 131.13, 131.75, 134.57, 134.96, 150.87; CH_2_ 61.94, COO 163.66, C = O 166.71.Anal. Calc. for C_16_H_10_N_2_O_6_: C, 58.90; H, 3.09; N, 8.59. Found: C, 58.98; H, 3.11; N, 8.58. CCDC No.: 1019344.

### Antioxidant evaluation

#### 2,2’-Diphenyl-1-picrylhydrazyl (DPPH) radical scavenging assay

All the test samples in addition to the standard antioxidant butylated hydroxytoluene (BHT) on DPPH radical scavenging was estimated according to the method reported [10]. Methanolic solution of the samples (10, 25, 50, 100, 200 and 500 μg/mL for samples; 0–5 μg/mL for BHT) in 200 μL aliquot was mixed with 100 mM tris-HCl buffer (800 μL, pH 7.4) and then added 1 mL of 500 μM DPPH in methanol (final concentration of 250 μM). The mixture was vigorously shaken and incubated in the dark at room temperature for 20 min. A DPPH blank solution (control) was prepared as above without the sample, and methanol was used for the baseline correction. The absorbance of the test solutions were measured spectro-photometrically at 517 nm. The DPPH radical scavenging activities were calculated using the equation:

DPPH radical scavenging activity (%) =[(Ac−As)/Ac] x 100

where, *Ac* is the absorbance of the control and *As* is the absorbance of the test samples. The inhibition concentration of the samples for 50% (IC_50_) DPPH radical scavenging was also calculated. Results were expressed as mean of the three determinations.

#### Ferric ion-reducing antioxidant power (FRAP) assay

All the synthesized compounds were evaluated for ferric reducing antioxidant property as described earlier by Oyaizu [11]. The theory behind this method is the reduction of ferric (Fe^3+^) to ferrous (Fe^2+^), which is accomplished in presence of antioxidants. Samples **4(a-n)** with the concentration of 10 μg/mL were mixed with equal volume of 0.2M phosphate buffer (pH 6.6) and 1% potassium ferricyanide and the mixture were incubated for 20 min at 50°C. Later the mixture was acidified with 2.5 mL of 10% trichloroacetic acid and centrifuged at 3000 rpm for about 15 min. The upper supernatant liquid was diluted with distilled water and 0.1% ferric chloride was added. The absorbance of this solution was measured at 700 nm. The increase in absorbance is directly proportional to the reducing ability of the tested samples. The control was prepared as above without the sample.

#### Cupric ion-reducing antioxidant capacity (CUPRAC) assay

The compounds were also tested for their cupric ion reducing property by the method reported [12]. CUPRAC is a widely applicable method for evaluating the antioxidant property of the substance. A mixture of CuCl_2_ (1 mL, 0.01 M) solution, ethanolic neocuproine (1 mL, 0.0075 M) and ammonium acetate (1 mL, 1 M) were dissolved and added 1 mL of test samples (10–50 μg/mL) along with 0.1 mL of distilled water. After 30 min of incubation, the mixture was measured at 450 nm against the blank solution. Control is prepared as above without the sample.

### Statistical analysis

All the assay measurement were performed in triplicate (n = 3) and are expressed as mean of the three determinations. The amount of compound required to inhibit DPPH free radicals by 50% (IC_50_) was graphically estimated using linear regression algorithm. Statistical significance was evaluated employing *t*-test and *P* < 0.05 which were considered to be significant.

## Results and Discussion

### Chemistry

The detailed synthetic routes adopted for the synthesis of the derivatives **4(a-n)** is depicted in Fig. 1. The intermediate **3** was synthesized according to the procedure reported [13, 14]. The derivatives **4(a-n)** was obtained by nucleophilic substitutions by benzoic acid derivatives, as per the reported procedure [15]. The target products **4(a-n)** were confirmed by analytical and spectral studies. Further, the compounds **4(a-n)** (except **4d, 4h** & **4m**) were also characterized by single crystal X-ray diffraction studies. All the synthesized compounds **4(a-n)** were evaluated for their *in-vitro* antioxidant properties.

The IR spectra of the phthalimide esters **4(a-n)** showed the absorption bands above 3000 cm^-1^ indicating unsaturation or the presence of C-H (benzene and isoindoline-3,5-dione) groups whereas the methylene group,-CH_2_, as well as methyl group,-CH_3_- revealed the asymmetric and symmetric C-H stretching frequencies near ≈2970 and ≈2840 cm^-1^ respectively [16]. Based on the infrared spectra studies, compounds **4(a-n)** also revealed the present of ν(C = C) and ν(C-N) bands which are usually found for benzene and isoindoline-3,5-dione groups. In addition, the **4(a-n)** compounds revealed two distinct ν(C = O) bands in the range of 1786–1725 cm^-1^; the ν(C = O) band with the higher wavelength number are attributed to the C = O of isoindoline-3,5-dione group and the ν(C = O) band with the lower wavelength number are attributed to the C = O of carboxylate anion [17–20]. The only exceptional cases are compounds **4(b-e)** revealed the present of ν(C-Cl) centering at ≈1071 cm^-1^ and compounds **4(l-n)** revealed the present of ν(NO_2_) centering at ≈1530 cm^-1^ respectively. The ^1^H NMR spectra of the phthalimide esters **4(a-n)** produced similarities to each other with the presence of-CH_2_- protons centering around δ≈ 5.96 ppm and revealed two well resolved sets of quartets centering around δ≈ 7.78 and 7.94 ppm with the integration values of 2:2, ascribed to the-CH- protons of isoindoline-3,5-dione group [19,20]. In addition, the benzene protons revealed different sets of multiplicity and integration values due to different position of substitution and these protons signals are located in the downfield region in the ^1^H NMR spectra. The only exceptional and predictable observation was the occurrence of the-CH_3_ and-OCH_3_ protons signals of compounds **4(f-k)** in the up-field region. Based on the integration values, the number of protons in compounds **4(a-n)** is in accordance with the number of protons proposed. ^13^C NMR spectrum of the phthalimide esters **4(a-n)** showed three distinct sets of carbon signals. In the downfield region, both δ(C = O) and δ(COO) signals are located at δ≈ 166.85 ppm and δ≈ 165.00 ppm respectively, whereas the-CH_2_- carbon signals are located in the up-field region centering around δ≈ 61.50 ppm [16–20]. The only exceptional and predictable observation was the occurrence of-CH_3_ and-OCH_3_ carbon signals of compounds **4(f-h)** and **4(i-k)** in the up-field region of ^13^C NMR spectra respectively. From the ^13^C NMR spectra study, the carbon signals of benzene and isoindoline-3,5-dione groups were found in the range of 112.12–163.82 ppm [16–20]. The carbons signals centering at δ≈ 124.10, 131.80 and 134.80 ppm were attributed to isoindoline-3,5-dione groups and the remaining signals were attributed to benzene carbons.

### X-ray crystallography

**Fig 2:**
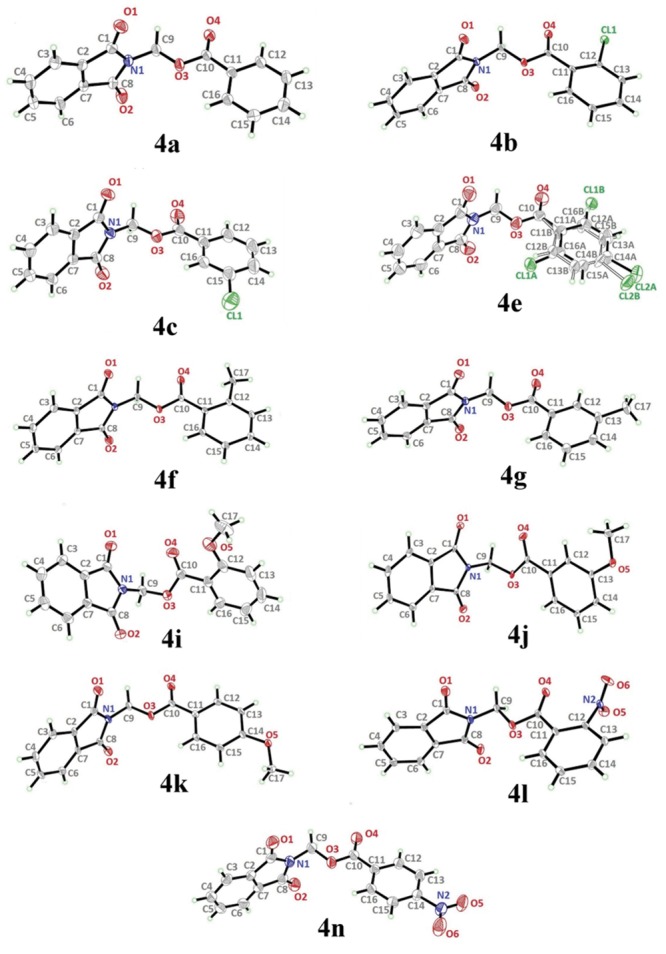
*ORTEP* diagram of compounds **4a** to **4n** (excluding 4d, 4h and 4m). Compounds **4b**, **4f**, **4g**, **4j**, **4k** and **4n** are drawn at 50% ellipsoids for non-hydrogen atoms and the remaining compounds are drawn at 30% ellipsoids for non-hydrogen atoms.

All the compounds were colourless and data were collected using MoKα radiation (λ = 0.71073 Å). The crystal structures of **4(a-n)** (excluding **4d**, **4h** and **4m**) are depicted in Fig. 2. Tables 1 & 2 give the crystallographic data and parameters for structure refinement. Table 3 lists the hydrogen bond geometries of these compounds. The dihedral angles and the torsion angles formed between the phthalimide and the benzene ring systems are shown in Table 4. Four out of the eleven compounds (**4a**, **4e**, **4g** and **4k**) were crystallized in monoclinic system with space group *P*2_1_/*c*. Whereas **4b**, **4f**, **4j** and **4l** crystallized in triclinic system with space group *P*-1 and the compounds **4i**, **4n** and **4c** crystallized in orthorhombic and tetragonal systems with space groups *Pbca*, *Pbcn* and *I*4_1_/*a*, respectively.

**Table 1:** Crystal data and parameters for structure refinement of 4a, 4b, 4c, 4e and 4f.

Compound	4a	4b	4c	4e	4f
CCDC number	1019340	1019275	1019341	1019342	1019276
Molecular formula	C_16_H_11_NO_4_	C_16_H_10_ClNO_4_	C_16_H_10_ClNO_4_	C_16_H_9_Cl_2_NO_4_	C_17_H_13_NO_4_
Molecular weight	281.26	315.70	315.70	350.14	295.28
Crystal system	Monoclinic	Triclinic	Tetragonal	Monoclinic	Triclinic
Space group	*P*2_1_/*c*	*P*-1	*I*4_1_/*a*	*P*2_1_/*c*	*P*-1
*a* (Å)	10.839 (1)	7.052 (2)	13.8967 (6)	14.340 (3)	7.3517 (5)
*b* (Å)	4.823 (1)	7.604 (2)	13.8967 (6)	13.951 (2)	7.6033 (5)
*c* (Å)	26.129 (3)	13.666 (6)	30.436 (3)	7.6331 (13)	13.658 (1)
α (°)	90	99.403 (1)	90	90	98.867 (2)
β (°)	106.372 (4)	98.632 (1)	90	103.005 (3)	99.506 (3)
γ (°)	90	107.496 (3)	90	90	110.979 (1)
*V* (Å^3^)	1310.6 (3)	673.89 (4)	5877.8 (8)	1487.9 (4)	683.93 (8)
*Z*	4	2	16	4	2
*D*_calc_ (g cm^−3^)	1.425	1.556	1.427	1.563	1.434
Crystal dimensions (mm)	0.56 × 0.17 × 0.12	0.55 × 0.41 × 0.08	0.43 × 0.33 × 0.16	0.57 × 0.19 × 0.09	0.54× 0.40× 0.39
μ (mm^−1^)	0.10	0.30	0.28	0.46	0.10
*T*_min_/*T*_max_	0.944/0.988	0.8577/0.9829	0.890/0.956	0.781/0.962	0.946 /0.961
Reflections measured	14810	24638	63100	16105	26359
Ranges/indices (*h, k, l*)	−13, 15; −6, 6; −36, 36	−10, 9; −10, 10; −19, 19	−10, 9; −10, 10; −19, 19	−19, 15; −19, 17; −10, 10	−10, 10; −10, 10; −19, 19
θ limit (°)	2.9–29.4	2.9–30.5	2.5–24.6	2.9–22.0	3.0–30.4
Unique reflections	3822	4129	4351	3964	4183
Observed reflections (I> 2σ(I))	2514	3332	2731	1900	3359
Paramters	190	199	199	257	200
Goodness of fit on *F* ^2^	1.04	1.05	1.02	0.99	1.07
*R*_1_, *wR*_2_ [*I* ≥ 2σ(*I*)]	0.043, 0.121	0.065, 0.188	0.046, 0.153	0.049, 0.199	0.050, 0.141

**Table 2:** Crystal data and parameters for structure refinement of 4g, 4i, 4j, 4k, 4l and 4n.

Compound	4g	4i	4j	4k	4l	4n
CCDC number	1019277	1019343	1019278	1019279	1019280	1019344
Molecular formula	C_17_H_13_NO_4_	C_17_H_13_NO_5_	C_17_H_13_NO_5_	C_17_H_13_NO_5_	C_16_H_10_N_2_O_6_	C_16_H_10_N_2_O_6_
Molecular weight	295.28	311.28	311.28	311.28	326.26	326.26
Crystal system	Monoclinic	Orthorhombic	Triclinic	Monoclinic	Triclinic	Orthorhombic
Space group	*P*2_1_/*c*	*Pbca*	*P*-1	*P*2_1_/*c*	*P*-1	*Pbcn*
*a* (Å)	4.3984 (2)	12.4243 (8)	8.4216 (5)	14.2125 (8)	7.5556 (5)	13.298 (2)
*b* (Å)	13.9960 (8)	7.1597 (5)	8.4471 (6)	13.0603 (8)	7.6118 (4)	7.549 (1)
*c* (Å)	22.487 (1)	32.917 (2)	10.8739 (7)	7.9266 (4)	13.6551 (8)	29.095 (3)
α (°)	90	90	80.311 (2)	90	99.132 (2)	90
β (°)	97.622 (2)	90	86.382 (2)	100.672 (2)	97.068 (2)	90
γ (°)	90	90	66.304 (2)	90	113.204 (2)	90
*V* (Å^3^)	1372.07 (12)	2928.1 (3)	698.22 (8)	1445.88 (14)	697.59 (7)	2920.6 (7)
*Z*	4	8	2	4	2	8
*D*_calc_ (g cm^−3^)	1.429	1.412	1.481	1.430	1.553	1.484
Crystal dimensions (mm)	0.70 × 0.07 × 0.06	0.52 × 0.46 × 0.18	0.46 × 0.40 × 0.16	0.57 × 0.35 × 0.11	0.56× 0.25× 0.16	0.70 × 0.07 × 0.06
μ (mm^−1^)	0.10	0.11	0.11	0.11	0.12	0.12
*T*_min_/*T*_max_	0.931/0.994	0.947/0.981	0.951/0.983	0.942/0.988	0.935 /0.981	0.930/0.991
Reflections measured	72106	32340	18754	38131	34125	17655
Ranges/indices (*h, k, l*)	−6, 6; −20, 20; −32, 32	−17, 17; −9, 10; −46, 46	−11, 12; −12, 12; −15, 15	−20, 20; −18, 18; −10, 11	10, 10; −10, 10; −19, 19	−15, 18; −10, 10; −40, 40
θ limit (°)	2.3–24.3	2.5–28.6	2.6–30.5	2.9–30.5	3.0–30.5	3.1–21.6
Unique reflections	4235	4405	4282	4446	4279	4242
Observed reflections (*I*> 2σ(*I*))	2400	3155	3198	2696	3327	1707
Parameters	200	209	209	209	217	217
Goodness of fit on *F* ^2^	1.00	1.03	1.02	1.00	1.04	0.93
*R*_1_, *wR*_2_ [*I* ≥ 2σ(*I*)]	0.061, 0.130	0.045, 0.138	0.068, 0.193	0.075, 0.211	0.047, 0.130	0.057, 0.213

**Table 3:** Hydrogen bond geometries for the compounds 4a-4n (excluding 4d, 4h and 4m).

*D*–H · · ·*A*	*d*(*D*–H) (Å)	*d*(H · · ·*A*) (Å)	*d*(*D*· · ·*A*) (Å)	*Angle* (*D*–H · · ·*A*) (°)
**4a**				
C3—H3A· · · O1^i^	0.93	2.46	3.354 (2)	162
C9—H9A · · · O4^ii^	0.97	2.59	3.300 (2)	130
**4b**				
C3—H3A· · · O1^iii^	0.93	2.51	3.382(3)	157
C6—H6A · · · O2^iv^	0.93	2.42	3.270(3)	151
C15—H15A · · · O4^v^	0.93	2.37	3.290(3)	169
**4c**				
C6—H6A · · · O1^vi^	0.93	2.40	3.317(2)	167
C3—H3A· · · O2^vii^	0.93	2.37	3.289(2)	168
C14—H14A · · · O4^viii^	0.93	2.36	3.111(3)	137
C16—H16A · · · O2^ix^	0.93	2.56	3.437(2)	157
**4e**				
C3—H3A· · · O2^x^	0.93	2.44	3.349 (4)	166
C6—H6A · · · O1^xi^	0.93	2.46	3.379 (3)	168
C15—H15A· · · O4^xii^	0.93	2.44	3.334 (6)	162
**4f**				
C3—H3A· · · O1^xiii^	0.93	2.36	3.256 (2)	163
C6—H6A· · · O2^xiv^	0.93	2.42	3.332 (2)	168
C15—H15A· · · O4^xv^	0.93	2.39	3.300 (2)	168
**4g**				
C3—H3A· · · O1^xvi^	0.93	2.49	3.393 (2)	164
**4j**				
C3—H3A· · · O4^xiv^	0.93	2.55	3.287 (2)	136
C17—H17A· · · O2 ^xvii^	0.96	2.50	3.340 (2)	146
**4k**				
C4—H4A· · · O5^xviii^	0.93	2.50	3.220 (3)	134
C15—H15A· · · O4^xii^	0.93	2.56	3.491 (3)	176
**4l**				
C3—H3A· · · O1^xix^	0.93	2.56	3.470 (2)	166
C5—H5A· · · O6^xx^	0.93	2.58	3.467 (2)	159
C6—H6A· · · O2^xxi^	0.93	2.41	3.304 (2)	161
C15—H15A · · · O4^xxii^	0.93	2.35	3.262 (2)	168
**4n**				
C12—H12A · · · O6^v^	0.93	2.45	3.363 (4)	167
C15—H15A · · · O4^xv^	0.93	2.36	3.274 (4)	166

Symmetry codes:

(i) −*x*, −*y*+3, −*z*+2;

(ii) −*x*, *y*+1/2, −*z*+3/2;

(iii) −*x*+1, −*y*+1, −*z*+1;

(iv) −*x*−1, −*y*, −*z*+1;

(v) *x*, *y*+1, *z*;

(vi) *x*−1/2, *y*, −*z*+1/2;

(vii) *x*+1/2, *y*, −*z*+1/2;

(viii) *x*, *y*−1/2, −*z*;

(ix) −*x*+1, −*y*+1/2, *z*;

(x) −*x*+1, *y*+1/2, −*z*−1/2;

(xi) −*x*+1, *y*−1/2, −*z*−1/2;

(xii) *x*, *y*, *z*+1;

(xiii) −*x*+3, −*y*+1, −*z*+1;

(xiv) −*x*+1, −*y*, −*z*+1;

(xv) *x*, *y*−1, *z*;

(xvi) −*x*−1, −*y*+1, −*z*+2;

(xvii) *x*+1, *y*−1, *z*;

(xviii) *x*−1, −*y*+3/2, *z*−1/2;

(xix)-*x*,-*y*,-*z*+1;

(xx) *x*, *y*+1, *z*+1;

(xxi)-*x*+1,-*y*+2,-*z*+1;

(xxii) *x*-1, *y*, *z*.

**Table 4:** Dihedral angles and torsion angles formed between the phthalimide and benzene ring systems.

Compound	Dihedral angle (°)	Torsion angle of C10—O3—C9—N1 (°)
**4a**	87.00 (6)	-176.16(11)
**4b**	86.39 (9)	170.31(14)
**4c**	85.05 (8)	-179.63(13)
**4e**	Part A 89.34; Part B 87.71	-175.81(19)
**4f**	86.06 (6)	-169.03(9)
**4g**	80.76 (8)	-177.95(15)
**4i**	79.86 (7)	-86.50(14)
**4j**	73.31 (7)	-91.92(15)
**4k**	83.32 (8)	-174.63(17)
**4l**	86.71 (6)	-171.84(10)
**4n**	83.91 (10)	166.2(2)

**Fig 3:**
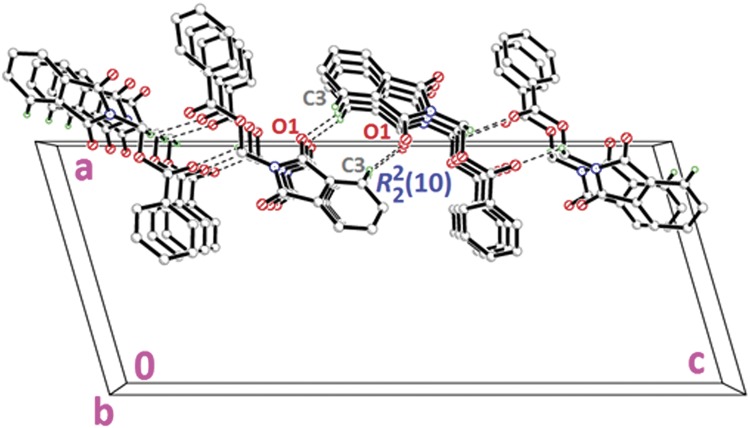
Crystal structure of **4a**, showing the sheet parallel to *bc*-plane, viewed along the *b*-axis.

**Fig 4:**
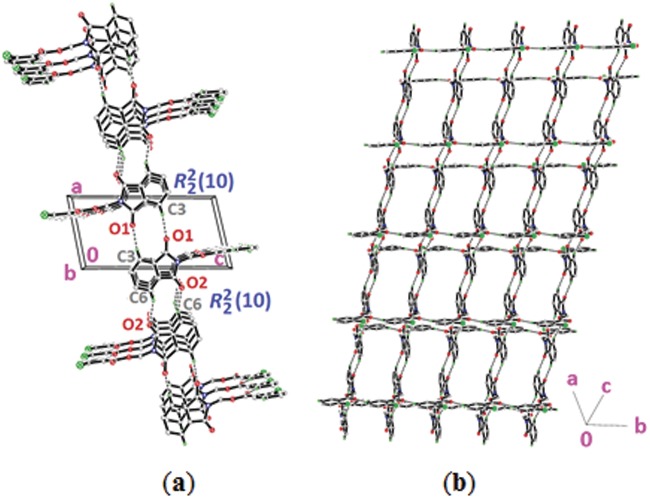
Crystal structure of **4b**, showing the formation of sheet parallel to a*b*-plane.

**Fig 5:**
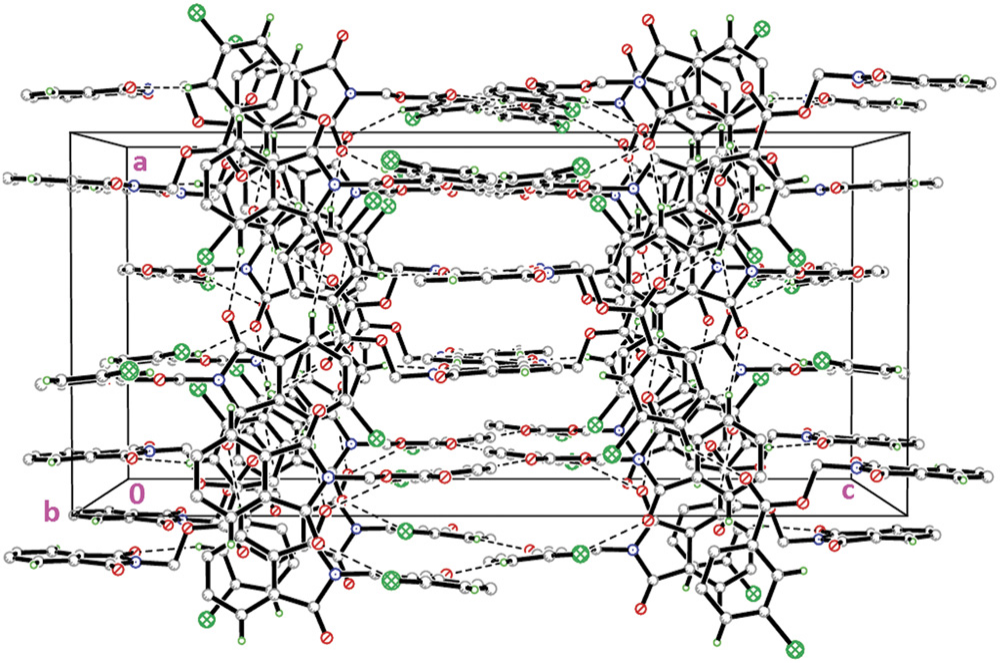
Crystal structure of **4c**, showing the three-dimensional network, viewed along the *b*-axis.

**Fig 6:**
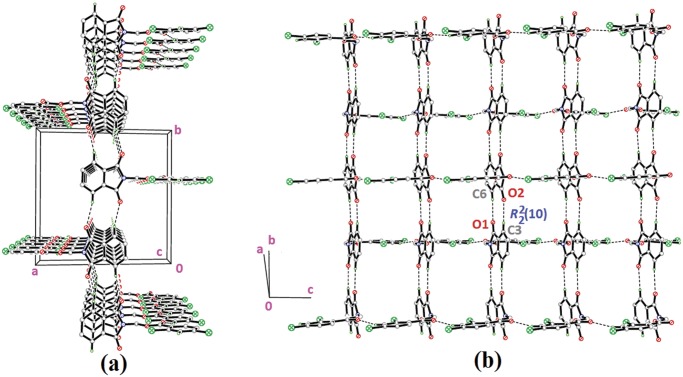
Crystal structure of **4e**, showing the ring motifs which lead to the formation of sheet parallel to *bc*-plane.

Compound **4a** with no substitution on the benzene ring is illustrated in Fig. 2(4a). The phthalimide (N1/C1—C8) and the benzene (C11—C16) rings are almost perpendicular to each other with the dihedral angle of 87.00 (6)° and the torsion angle between C10—O3—C9—N1 being-176.16 (11)° (Table 4). Compound **4a** is considered as a parental skeleton for the comparison of compounds with varrying substitutions on the benzene ring. In the crystal structure (Fig. 3), molecules are linked into dimers *via* intermolecular C3—H3A···O1 hydrogen bonds (Table 3), forming $R_{2}^{2}(10)$ring motif [20] and are further connected into sheets parallel to *bc*-plane by intermolecular C9—H9A···O4 hydrogen bonds (Table 3). Compounds **4b** and **4c** exist with one—chloro substituent at—*ortho* and—*para* positions of the benzene rings (Fig. 2(4b & 4c)), respectively, whereas compound **4e** (Fig. 2(4e)) differs by having—dichloro substituents at–*ortho* and—*meta* positions of the benzene ring. The dichloro-benzene ring in compound **4e** is disordered over two positions with the occupancy ratio of 0.791 (2): 0.219 (2). In the crystal structure of **4b** (Fig. 4a), intermolecular C3—H3A···O1 and C6—H6A···O2 hydrogen bonds (Table 3) link the molecules to form their respective dimers with $R_{2}^{2}(10)$ring motifs [20]. In compounds **4c** and **4e**, the adjacent molecules are connected to form $R_{2}^{2}(10)$ring motifs [20] through intermolecular C3—H3A···O2 and C6—H6A···O1 hydrogen bonds (Table 3, Figs. 5 & 6a). Further, the molecules in compounds **4b** and **4e** are linked into sheets *via* intermolecular C15—H15A···O4 hydrogen bonds (Table 3) extending parallel to *ab* and *bc*-plane for **4b** and **4e**, respectively (Fig. 4b & Fig. 6b). The crystal packing of **4b** is further consolidated by π···π interactions between the five-membered ring (N1/C1/C2/C7/C8; centroid *Cg*1) and benzene ring (C2—C7; centroid *Cg*2), where the separations of centroids of *Cg*1···*Cg*1, *Cg*1···*Cg*2 and *Cg*2···*Cg*2 being 3.4860 (13), 3.6022 (13) and 3.5996 (12) Å, respectively. Whereas, a three-dimensional network involving the ring motifs was produced by the C14—H14A···O4 and C16—H16A···O2 intermolecular hydrogen bonds in compound **4c** (Fig. 6).

**Fig 7:**
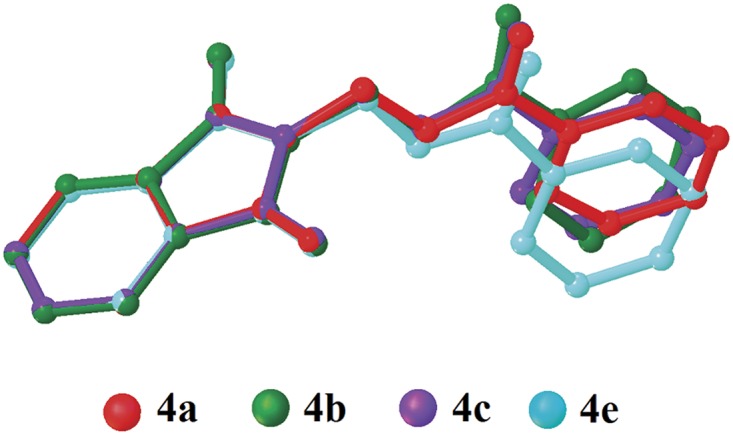
Overlay of all non-H atoms in compounds **4a, 4b, 4c** and **4e**, calculated using the phthalimide moiety.

The dihedral angles between the phthalimide and the benzene rings (Table 4) of **4b**, **4c** and **4e** indicate that, they lie perpendicular to each other in the range of 85.05 (8) to 89.34° and the C10—O3—C9—N1 torsion angles fall within the range of 170.31 (14) to-179.63 (13)°. Fig. 7 shows the overlaid molecules over all non-H atoms, calculated using the phthalimide moiety with the r.m.s values of 0.095 Å for **4a**/**4b**, 0.141 Å for **4a**/**4c** and 0.350 Å for **4a**/**4e**.

**Fig 8:**
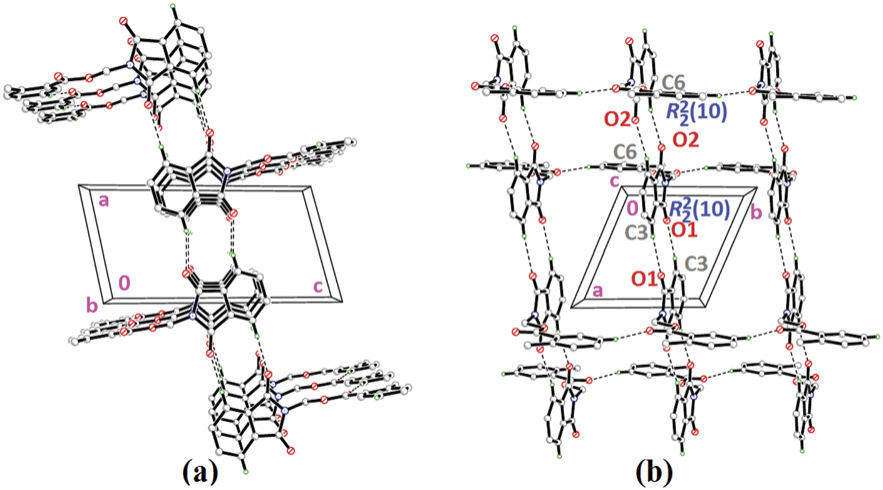
Crystal structure of **4f**, showing the dimers which are further linked into sheet parallel to a*b*-plane.

**Fig 9:**
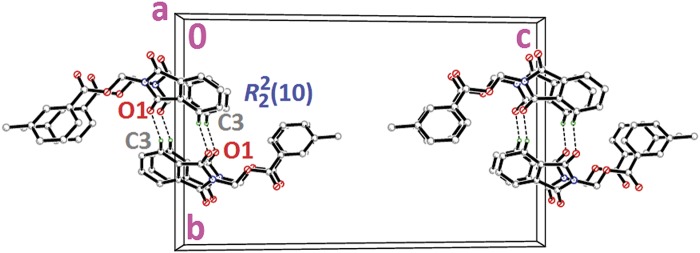
Crystal structure of **4g**, showing the formation of dimers stacked along the *a*-axis.

**Fig 10:**
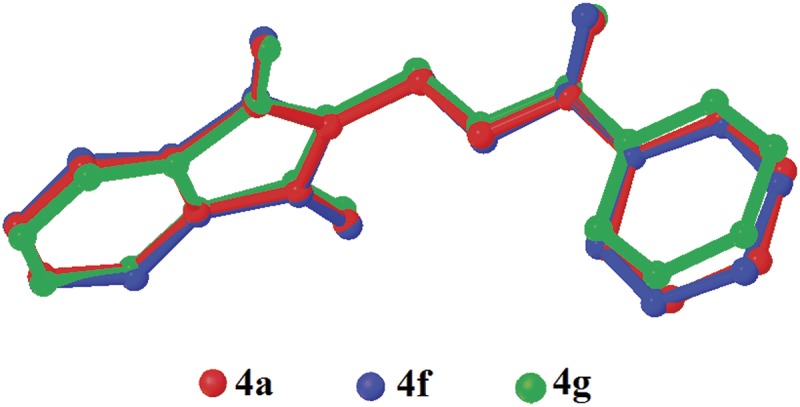
Overlay of all non-H atoms in compounds **4a, 4f** and **4g**, calculated using the phthalimide moiety.

Compounds **4f** and **4g** (Fig. 2(4f & 4g)) consist one methyl substituent at—*ortho* and—*meta* positions, respectively. In the crystal structure of **4f** (Fig. 8a), dimers were formed by intermolecular C3—H3A···O1 and C6—H6A···O2 hydrogen bonds (Table 3) at an alternate fashion, giving $R_{2}^{2}(10)$ring motifs [20]. These set of ring motifs were then further linked into planes through intermolecular C15—H15A···O4 hydrogen bonds (Table 3) parallel to the *ab*-plane (Fig. 8b) and π···π interactions stabilized the crystal structure with the separation of centroid-centroid, *Cg*1···*Cg*1, *Cg*1···*Cg*2 and *Cg2*···*Cg*2 being 3.4930 (8), 3.5902 (8) and 3.6003 (8) Å. Intermolecular C3—H3A···O1 hydrogen bonds (Table 3) joined the molecules in **4g** into dimers as depicted in Fig. 9, forming graph set notation of $R_{2}^{2}(10)$ring motifs [21]. The conformation of both the compounds are very analogous to each other with the dihedral angles between the rings being 86.06 (6) and 80.76 (8)° and their C10—O3—C9—N1 torsion angles are-169.03 (9) and-177.95 (15)°, respectively (Table 4). The analogous conformations is best visualized by the overlay (Fig. 10) of **4a**, **4f** and **4g**, calculated using phthalimide ring system, with the r.m.s values of 0.094 Å for **4a**/**4f** and 0.156 Å for **4a**/**4g**.

**Fig 11:**
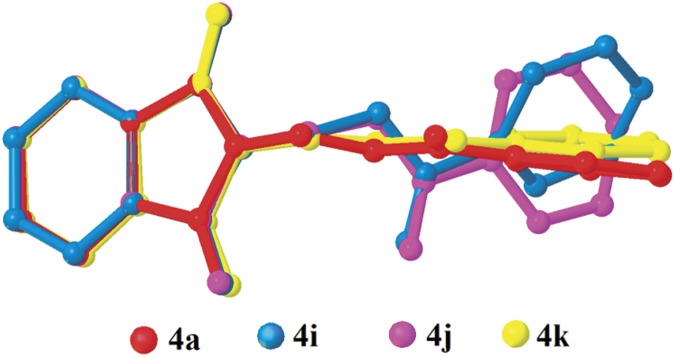
Overlay of all non-H atoms in compounds **4a, 4i, 4j** and **4k**, calculated using the phthalimide moiety.

**Fig 12:**
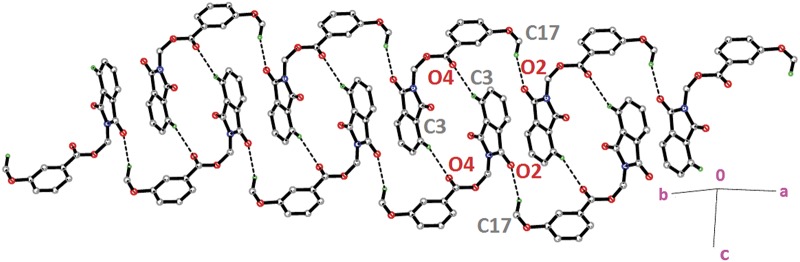
Crystal structure of **4j**, showing the column formed along [–110] *via* intermolecular C—H···O hydrogen bonds.

**Fig 13:**
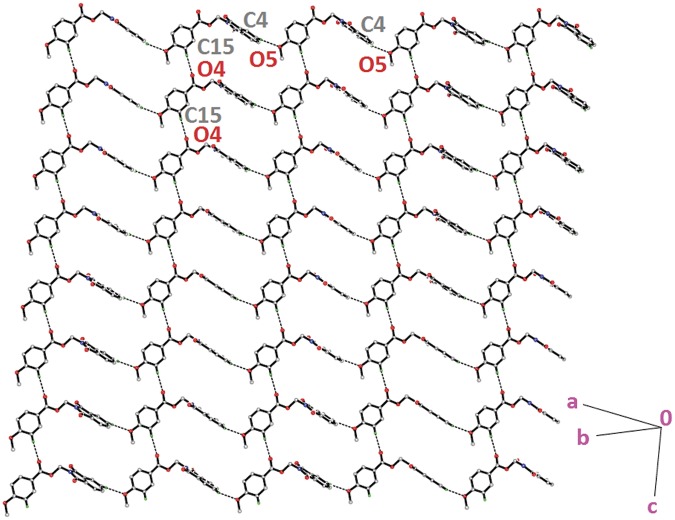
Crystal structure of **4k**, showing the sheet formed parallel to *ac*-plane *via* intermolecular C—H···O hydrogen bonds.

Compounds **4i**, **4j** and **4k** (Fig. 2(4i-4k)) contain-methoxy substitution at—*ortho*,—*meta* and—*para* positions on the benzene rings, respectively. The 79.86 (7) and 73.31 (7)° dihedral angles between phthalimide and benzene rings for compounds **4i** and **4j** respectively, suggest that, they have slightly different conformation from the parental skeletal **4a**. This is confirmed by the C10—O3—C9—N1 torsion angles being-86.50 (14) and-91.92 (15)°, respectively for **4i** and **4j**. While, the respective parameters for compound **4k** are 83.32 (8) and-174.63 (17)°. This conformation difference of the compounds **4i** and **4j** compared with **4a** and **4k** can be clearly viewed in Fig. 11, which shows the overlays of all non-H atoms of **4a/4i**, **4a/4j** and **4a/4k**, calculated using the phthalimide moiety where their H-atoms were excluded, with the r.m.s values of 0.823, 0.895 and 0.154 Å, respectively. There is no significant intermolecular hydrogen bond observed in **4i**. However, π···π interactions are observed to stabilize the crystal structure, involving the phthalimide ring system and the phenyl ring, with *Cg*1···*Cg*2 being 3.6764 (10) Å and *Cg*3···*Cg*3 being 3.6449 (9) Å, where Cg3 is the centroid of C11—C17. Fig. 12 illustrates the molecular packing of **4j**. Intermolecular C3—H3A···O4 hydrogen bond (Table 3) links the adjacent molecules in **4j** to form dimers, giving $R_{2}^{2}(10)$ring motifs [21]. These set of dimers were then connected into columns along [–110] by intermolecular C17—H17A···O2 hydrogen bond (Table 3). The crystal structure of **4k** is depicted in Fig. 13. The molecules in **4k** are linked into chains along the *a-*axis by intermolecular C4—H4A···O5 hydrogen bonds (Table 3) and are further connected into sheets parallel to *ac*-plane by intermolecular C15—H15A···O4 hydrogen bonds (Table 3).

**Fig 14:**
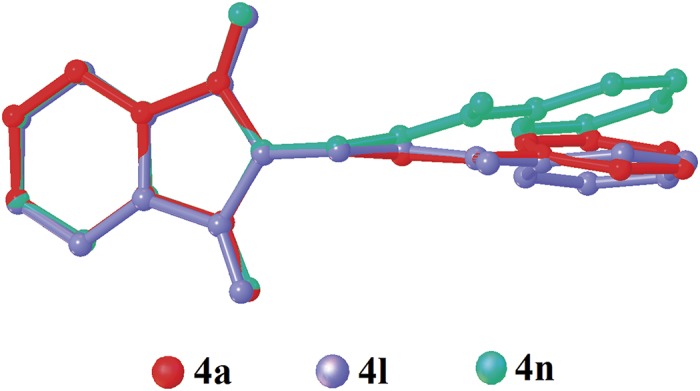
Overlay of all non-H atoms in compounds **4a, 4l** and **4n**, calculated using the phthalimide moiety.

**Fig 15:**
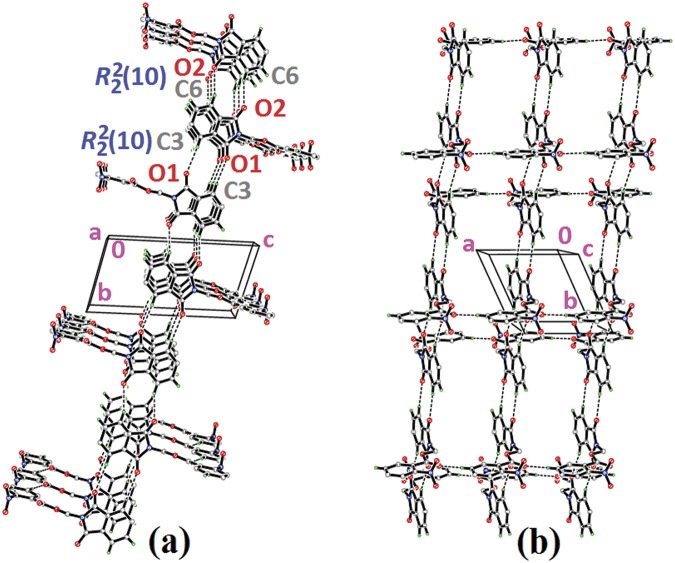
Crystal structure of **4l**, showing the dimers which are further connected to form sheet parallel to a*b*-plane.

**Fig 16:**
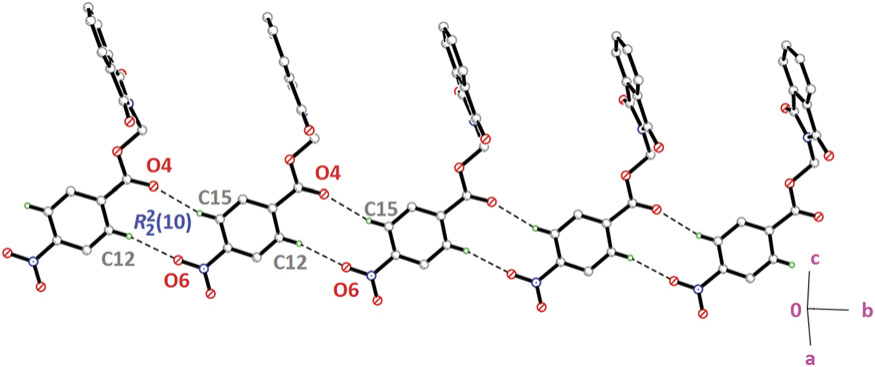
Crystal structure of **4n**, showing the ring motifs which are linked into chain along *b*-axis *via* intermolecular C—H···O hydrogen bonds.

The molecular structures of compounds with nitro-substituent at—*ortho* and—*para* positions on the benzene rings, **4l** and **4n** are shown in Fig. 2(4l & 4n). The dihedral angles formed between the ring members are 86.71 (6)° for **4l** and 83.91 (10)° for **4n**. The C10—O3—C9—N1 torsion angle of **4l** is-171.84 (10)°, whereas the respective parameter for **4n** is 166.2 (2)°. These two compounds exist with the similar conformation as indicated by the overlay structures in Fig. 14. The overlays of all non-H atoms of **4a/4l** and **4a/4n**, calculated using the phthalimide moiety where their H-atoms and nitro-substituent were excluded, with the r.m.s values of 0.117 and 0.096 Å, respectively. In the crystal structure of **4l**, intermolecular C3—H3A···O1 and C6—H6A···O2 hydrogen bonds link the adjacent molecules to form their respective dimers (Fig. 15a) alternately, with $R_{2}^{2}(10)$ring motifs [21]. These set of dimers were then further connected into sheets parallel to *ab*-plane *via* intermolecular C5—H5A···O6 and C15—H15A···O4 hydrogen bonds as shown in Fig. 15b. The crystal structure of **4n** is shown in Fig. 16. Intermolecular C12—H12A···O6 and C15—H15A···O4 hydrogen bonds link the neighbouring molecules into chains along the *b*-axis, forming $R_{2}^{2}(10)$ring motifs [21]. Both **4l** and **4n** are observed to have π···π interactions in their crystal packing. In compound **4l**, *Cg*1···*Cg*2 and *Cg*2···*Cg*2 with the separations of centroid-centroid being 3.6443 (10) and 3.6076 (9) Å, respectively, consolidated the crystal packing. Whereas in compound **4n**, only *Cg*1···*Cg*2 with the separation of 3.5571 (17) Å is responsible for its crystal packing.

**Fig 17:**
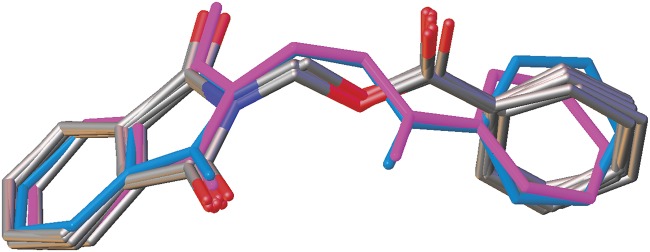
Overlay of all molecular structures, excluding H atoms and substituents at benzene rings where **4i** and **4j** are highlighted in blue and pink, respectively.

The overall conformations of the reported structures are described focused onto the dihedral and torsion angles formed between the phthalimide (N1/C1—C8) and the benzene (C11—C16) ring systems. The molecular structures of the compounds **4a-n** (excluding **4d**, **4h** and **4m**) were overlaid as shown in Fig. 17, excluding H atoms and substituents at the benzene rings. From the overlay diagram, it is clear that, the molecular conformations of majority of the compounds are almost similar to each other. Whereas the compounds **4i** and **4j** with-methoxy substituent at the—*ortho* and—*meta* position on the benzene rings are very much deviated from the parental skeletal (compound **4a)**.

## Antioxidant Activity

The synthesized compounds were screened for their antioxidant potential by employing the *in-vitro* assay such as DPPH free radical scavenging assay. The synthesized compounds were further tested for their reducing power ability. Ferric ion reducing antioxidant power (FRAP) assay and cupric ion reducing antioxidant capacity (CUPRAC) were measured using butylated hydroxytoluene as the standard.

### DPPH Radical Scavenging Assay

**Fig 18:**
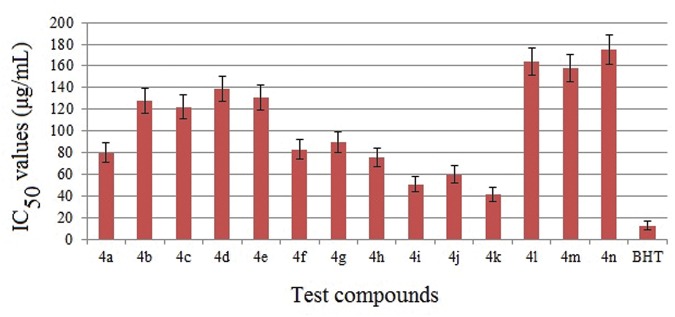
IC_50_ (concentration required for 50% inhibition) values for DPPH radical scavenging activities of the compounds **4(a–n)** in comparison with the standard antioxidant BHT. Lower IC_50_ values indicate higher radical scavenging activity.

The DPPH radical scavenging test is a standard and widely used assay for *in vitro* antioxidant capacity of compounds and it is based on their ability of scavenging of stable 1,1-diphenyl-2-picrylhydrazyl radical (DPPH) [10]. The results of *in vitro* antioxidant activity (IC_50_ values) of the synthesized compounds in comparison with the reference antioxidant BHT are depicted in Fig. 18. From the results, it is noticed that these compounds exhibit mild to good antioxidant activity. At the start with concentrations of 10–50 μg/mL, no significant change in the radical scavenging was noticed. However, the radical scavenging ability increased with increasing concentration of the samples (100–500 μg/mL). The IC_50_ values indicate that the compounds **4i**, **4j** and **4k** with—methoxy substitution at the—*o*,—*m* &—*p* position, respectively, on the phenyl ring showed good free radical scavenging activity. Among those, compound **4k** with a *p*-methoxy substituent was more potent in free radical scavenging compared to the other test compounds. The compounds **4(f-h)**, bearing—methyl substitutions displayed moderate scavenging activity. The IC_50_ value for the compound **4a** with no substitution is in between to the **4(i-k)** and **4(f-g)** compounds. The compounds **4b**, **4c**, **4d** and **4e** bearing electronegative—chloro substitution were mild in their activity, while the compounds **4(l–n)** with electron withdrawing—nitro substitutions respectively displayed very mild scavenging activity. Among them, compound **4n** exhibited the lowest activity. The biological data suggested that the substituents on the phenyl group had a profound effect on the antioxidant activity in the order of NO_2_ < Cl < H < CH_3_ < OCH_3_, which is consistent with those reported earlier [22].

### FRAP Assay

**Fig 19:**
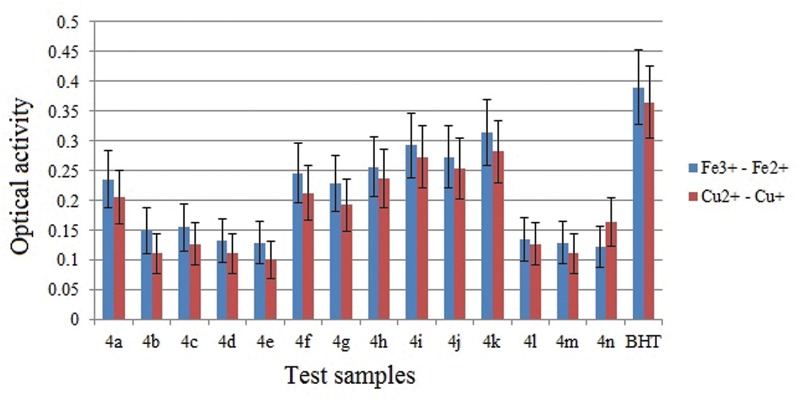
Ferric and cupric ion reducing power abilities of the tested samples at 10 μg concentration measured at 700 nm. Values are expressed as absorbance; high absorbance indicates high reducing power

In general, the antioxidant activity of a substance is directly correlated to its reducing ability. Standard assays like FRAP provide a reliable method to verify the antioxidant ability of a substance. Ferric reducing antioxidant properties for the synthesized compound were evaluated by the method as described earlier by Oyaizu [11]. Substances having reduction potential react with potassium ferricyanide forming potassium ferrocyanide. The formed potassium ferrocyanide further reacts with FeCl_3_ to form an intense Prussian blue complex which has a maximum absorbance at 700 nm. The complex formed is directly proportional to the reducing capacity of the test sample. An increase in absorbance is equal to the reducing power of the sample. Results are depicted in Fig. 19 and from the analysis, it is clear that the compounds **4i**, **4j**, **4k** with—methoxy substitution showed good cupric reducing ability, while the compounds **4a** with no substitution, **4(f-h)** with—methyl substitutions were moderate, and the compounds **4b**, **4c**, **4d**, **4e**, **4l**, **4m** and **4n** bearing—chloro and—nitro substitutions showed the lowest reducing ability among the series. This was in agreement with the similar reported structures [22].

### CUPRAC Assay

The cupric ion reducing properties of the synthesized compounds were evaluated by the method reported earlier [12]. In this assay a sample under evaluation effectively reduces Cu^2+^ to Cu^+^, changing the characteristic ion absorption. The reduced Cu+ ion combines with the chromogenic reagent neocuproine forming a stable 2:1 complex which has a maximum absorption at 450 nm. This method operates at pH 7. Results, shown in Fig. 19, indicate that the majority of these test compounds have good reducing ability. These compounds displayed 40% less reducing power compared to the standard. Compounds **4i**, **4j**, **4k** showed good cupric reducing ability, while the compounds **4a**, **4f**, **4g** and **4h** were moderate, and the compounds **4b**, **4c**, **4d**, **4e**, **4l**, **4m** and **4n** showed the lowest reducing ability among the series. The similar trend of increasing antioxidant activity was also observed in the related structures [22].

## Conclusion

Herein we describe the efficient synthesis of *N*-ethyl phthalimide esters **4(a-n)**. The synthesized compounds were confirmed from their spectral data. The conformations of the compounds **4(a-n)** (excluding **4d**, **4h** and **4m**) were confirmed by single crystal X-ray diffraction analysis. The molecular conformations of the reported compounds were compared among each other and found that, the compounds **4i** and **4j** deviate from the rest. The X-ray data also revealed the importance of intermolecular and other minor interactions contributing for the crystal structure stability and molecular packing, induced by varying substituent(s) on the phenyl ring. The synthesized compounds were evaluated for their *in vitro* antioxidant activities. Among the series, compounds **4i**, **4j** and **4k** with—methoxy substitution at—*ortho*,-*meta* and—*para* positions respectively displayed good antioxidant properties. However, the incorporation of electron releasing or electron withdrawing groups on the phthalimide ring system may result in better activities.

## Supporting Information

S1 Supporting Information CheckCIF report of compounds 4(a-n) (excluding 4d, 4h and 4m). (PDF)
