# Supplementary material for: Synthesis, Structural and Antioxidant Studies of Some Novel N-Ethyl Phthalimide Esters
Source: PLoS One. 2015 Mar 5;10(3):e0119440. doi: 10.1371/journal.pone.0119440 (PMC4351070; doi:10.1371/journal.pone.0119440)

checkCIF/PLATON report

Structure factors have been supplied for datablock(s) mo_ph1a_0m

THIS REPORT IS FOR GUIDANCE ONLY. IF USED AS PART OF A REVIEW PROCEDURE FOR PUBLICATION, IT SHOULD NOT REPLACE THE EXPERTISE OF AN EXPERIENCED CRYSTALLOGRAPHIC REFEREE.

No syntax errors found.   CIF dictionary   Interpreting this report

Datablock: mo_ph1a_0m

```
Bond precision:  C-C = 0.0020 A         Wavelength=0.71073

Cell:            a=10.8389(14)     b=4.8233(6)        c=26.129(3)
                 alpha=90          beta=106.372(4)    gamma=90
Temperature:     297 K

                 Calculated                 Reported
Volume           1310.6(3)                  1310.6(3)
Space group      P 21/c                     P 21/c
Hall group       -P 2ybc                    -P 2ybc
Moiety formula   C16 H11 N O4               C16 H11 N O4
Sum formula      C16 H11 N O4               C16 H11 N O4
Mr               281.26                     281.26
Dx,g cm-3        1.425                      1.425
Z                4                          4
Mu (mm-1)        0.104                      0.104
F000             584.0                      584.0
F000'            584.32
h,k,lmax         15,6,36                    15,6,36
Nref             3840                       3822
Tmin,Tmax        0.979,0.988                0.944,0.988
Tmin'            0.943

Correction method= MULTI-SCAN

Data completeness= 0.995          Theta(max)= 30.040

R(reflections)= 0.0433( 2514)     wR2(reflections)= 0.1213( 3822)

S = 1.044                    Npar= 190
```

The following ALERTS were generated. Each ALERT has the format
test-name_ALERT_alert-type_alert-level.
Click on the hyperlinks for more details of the test.

Alert level C

```
PLAT911_ALERT_3_C Missing # FCF Refl Between THmin & STh/L=  0.600         15 Report
```

Alert level G

```
PLAT128_ALERT_4_G Alternate Setting for Input Space Group  P21/c       P21/n Note
PLAT912_ALERT_4_G Missing # of FCF Reflections Above STh/L=  0.600          4 Note
```

```
   0 ALERT level A = Most likely a serious problem - resolve or explain
   0 ALERT level B = A potentially serious problem, consider carefully
   1 ALERT level C = Check. Ensure it is not caused by an omission or oversight
   2 ALERT level G = General information/check it is not something unexpected

   0 ALERT type 1 CIF construction/syntax error, inconsistent or missing data
   0 ALERT type 2 Indicator that the structure model may be wrong or deficient
   1 ALERT type 3 Indicator that the structure quality may be low
   2 ALERT type 4 Improvement, methodology, query or suggestion
   0 ALERT type 5 Informative message, check
```

It is advisable to attempt to resolve as many as possible of the alerts in all categories. Often the minor alerts point to easily fixed oversights, errors and omissions in your CIF or refinement strategy, so attention to these fine details can be worthwhile. In order to resolve some of the more serious problems it may be necessary to carry out additional measurements or structure refinements. However, the purpose of your study may justify the reported deviations and the more serious of these should normally be commented upon in the discussion or experimental section of a paper or in the "special_details" fields of the CIF. checkCIF was carefully designed to identify outliers and unusual parameters, but every test has its limitations and alerts that are not important in a particular case may appear. Conversely, the absence of alerts does not guarantee there are no aspects of the results needing attention. It is up to the individual to critically assess their own results and, if necessary, seek expert advice.

Publication of your CIF in IUCr journals

A basic structural check has been run on your CIF. These basic checks will be run on all CIFs submitted for publication in IUCr journals (*Acta Crystallographica*, *Journal of Applied Crystallography*, *Journal of Synchrotron Radiation*); however, if you intend to submit to *Acta Crystallographica Section C* or *E*, you should make sure that full publication checks are run on the final version of your CIF prior to submission.

Publication of your CIF in other journals

Please refer to the *Notes for Authors* of the relevant journal for any special instructions relating to CIF submission.

PLATON version of 24/07/2014; check.def file version of 24/07/2014

Datablock mo_ph1a_0m - ellipsoid plot

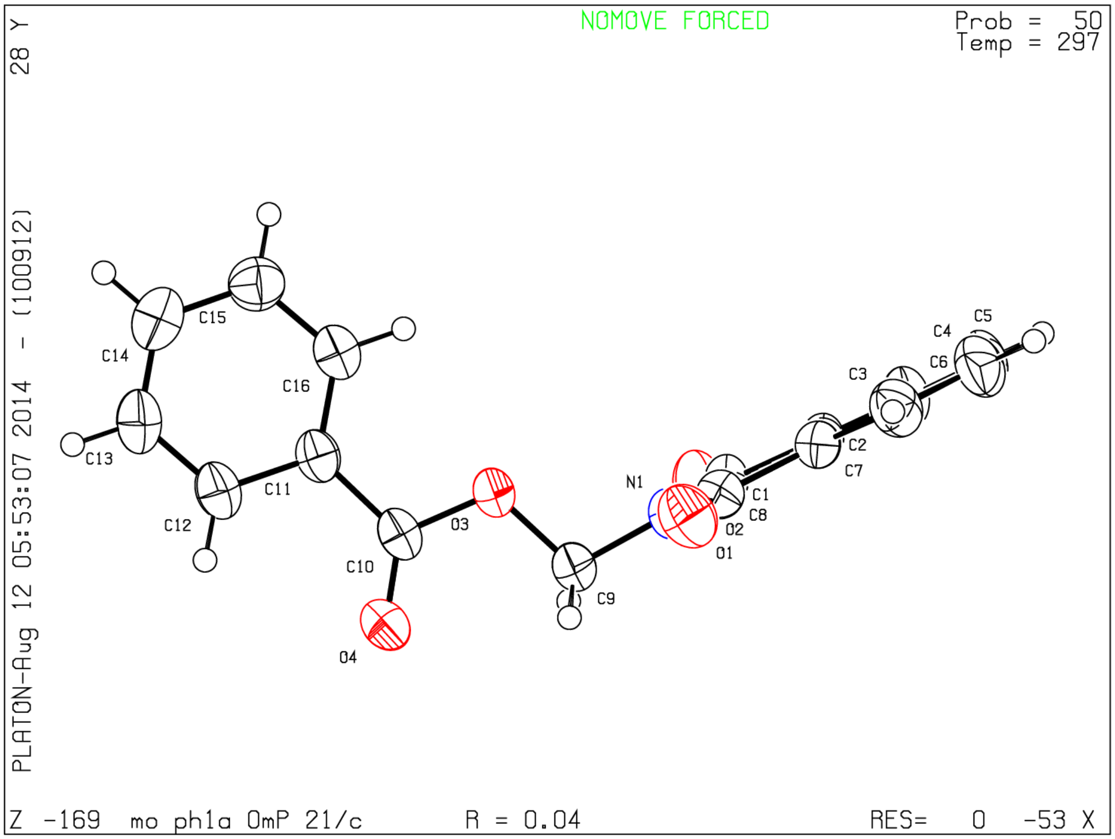

checkCIF/PLATON report

Structure factors have been supplied for datablock(s) mo_ph2a_0m

THIS REPORT IS FOR GUIDANCE ONLY. IF USED AS PART OF A REVIEW PROCEDURE FOR PUBLICATION, IT SHOULD NOT REPLACE THE EXPERTISE OF AN EXPERIENCED CRYSTALLOGRAPHIC REFEREE.

No syntax errors found. CIF dictionary Interpreting this report

Datablock: mo_ph2a_0m

```
Bond precision:  C-C = 0.0030 A          Wavelength=0.71073

Cell:          a=7.0522(2)       b=7.6037(2)      c=13.6660(6)
               alpha=99.403(1)   beta=98.632(1)   gamma=107.496(3)
Temperature:   100 K

                Calculated                Reported
Volume          673.89(4)                 673.89(4)
Space group     P -1                      P -1
Hall group      -P 1                      -P 1
Moiety formula  C16 H10 Cl N O4           C16 H10 Cl N O4
Sum formula     C16 H10 Cl N O4           C16 H10 Cl N O4
Mr              315.70                    315.70
Dx,g cm-3       1.556                     1.556
Z               2                         2
Mu (mm-1)       0.302                     0.302
F000            324.0                     324.0
F000'           324.46
h,k,lmax        10,10,19                  10,10,19
Nref            4132                      4129
Tmin,Tmax       0.862,0.976               0.851,0.976
Tmin'           0.847

Correction method= MULTI-SCAN

Data completeness= 0.999          Theta(max)= 30.600

R(reflections)= 0.0651( 3332)     wR2(reflections)= 0.1883( 4129)

S = 1.053                  Npar= 199
```

The following ALERTS were generated. Each ALERT has the format
test-name_ALERT_alert-type_alert-level.
Click on the hyperlinks for more details of the test.

Alert level C

```
PLAT975_ALERT_2_C Check Calcd Residual Density  0.96A From      O4       0.62 eA-3
```

Alert level G

```
PLAT066_ALERT_1_G Predicted and Reported Tmin&Tmax Range Identical         ? Check
PLAT072_ALERT_2_G SHELXL First  Parameter in WGHT Unusually Large.      0.12 Report
PLAT910_ALERT_3_G Missing # of FCF Reflections Below Th(Min) .....         1 Report
```

```
   0 ALERT level A = Most likely a serious problem - resolve or explain
   0 ALERT level B = A potentially serious problem, consider carefully
   1 ALERT level C = Check. Ensure it is not caused by an omission or oversight
   3 ALERT level G = General information/check it is not something unexpected

   1 ALERT type 1 CIF construction/syntax error, inconsistent or missing data
   2 ALERT type 2 Indicator that the structure model may be wrong or deficient
   1 ALERT type 3 Indicator that the structure quality may be low
   0 ALERT type 4 Improvement, methodology, query or suggestion
   0 ALERT type 5 Informative message, check
```

It is advisable to attempt to resolve as many as possible of the alerts in all categories. Often the minor alerts point to easily fixed oversights, errors and omissions in your CIF or refinement strategy, so attention to these fine details can be worthwhile. In order to resolve some of the more serious problems it may be necessary to carry out additional measurements or structure refinements. However, the purpose of your study may justify the reported deviations and the more serious of these should normally be commented upon in the discussion or experimental section of a paper or in the "special_details" fields of the CIF. checkCIF was carefully designed to identify outliers and unusual parameters, but every test has its limitations and alerts that are not important in a particular case may appear. Conversely, the absence of alerts does not guarantee there are no aspects of the results needing attention. It is up to the individual to critically assess their own results and, if necessary, seek expert advice.

Publication of your CIF in IUCr journals

A basic structural check has been run on your CIF. These basic checks will be run on all CIFs submitted for publication in IUCr journals (*Acta Crystallographica*, *Journal of Applied Crystallography*, *Journal of Synchrotron Radiation*); however, if you intend to submit to *Acta Crystallographica Section C* or *E*, you should make sure that full publication checks are run on the final version of your CIF prior to submission.

Publication of your CIF in other journals

Please refer to the *Notes for Authors* of the relevant journal for any special instructions relating to CIF submission.

PLATON version of 24/07/2014; check.def file version of 24/07/2014

Datablock mo_ph2a_0m - ellipsoid plot

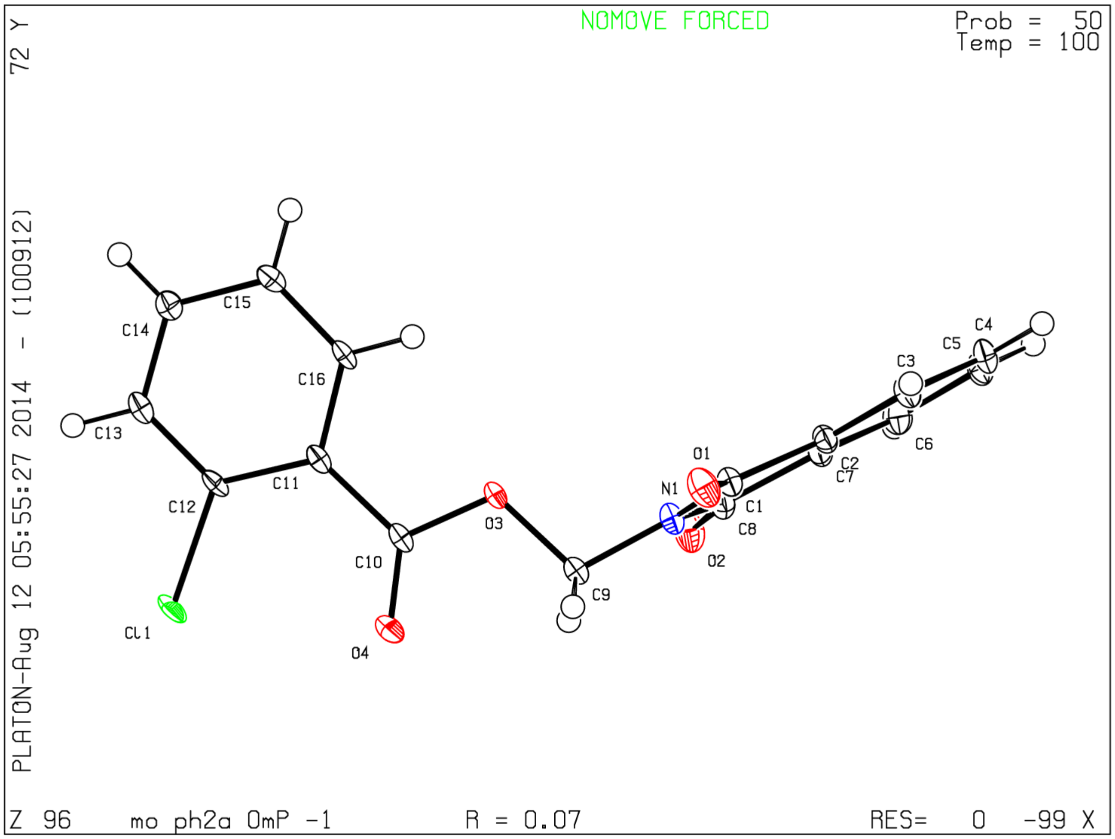

checkCIF/PLATON report

Structure factors have been supplied for datablock(s) mo_ph3_0m

THIS REPORT IS FOR GUIDANCE ONLY. IF USED AS PART OF A REVIEW PROCEDURE FOR PUBLICATION, IT SHOULD NOT REPLACE THE EXPERTISE OF AN EXPERIENCED CRYSTALLOGRAPHIC REFEREE.

No syntax errors found. CIF dictionary Interpreting this report

Datablock: mo_ph3_0m

```
Bond precision:  C-C = 0.0027 A          Wavelength=0.71073

Cell:             a=13.8967(6)      b=13.8967(6)      c=30.436(3)
                  alpha=90          beta=90           gamma=90
Temperature:      297 K

                Calculated                Reported
Volume          5877.8(8)                 5877.8(8)
Space group     I 41/a                    I 41/a
Hall group      -I 4ad                    -I 4ad
Moiety formula  C16 H10 Cl N O4           C16 H10 Cl N O4
Sum formula     C16 H10 Cl N O4           C16 H10 Cl N O4
Mr              315.70                    315.70
Dx,g cm-3       1.427                     1.427
Z               16                        16
Mu (mm-1)       0.277                     0.277
F000            2592.0                    2592.0
F000'           2595.67
h,k,lmax        19,19,42                  19,19,42
Nref            4353                      4351
Tmin,Tmax       0.896,0.957               0.890,0.956
Tmin'           0.888

Correction method= MULTI-SCAN

Data completeness= 1.000          Theta(max)= 30.150

R(reflections)= 0.0461( 2731)     wR2(reflections)= 0.1530( 4351)

S = 1.025                  Npar= 199
```

The following ALERTS were generated. Each ALERT has the format
test-name_ALERT_alert-type_alert-level.
Click on the hyperlinks for more details of the test.

Alert level C

```
PLAT905_ALERT_3_C Negative K value in the Analysis of Variance ...     -4.576 Report
```

Alert level G

```
PLAT066_ALERT_1_G Predicted and Reported Tmin&Tmax Range Identical          ? Check
PLAT912_ALERT_4_G Missing # of FCF Reflections Above STh/L=  0.600          3 Note
```

```
   0 ALERT level A = Most likely a serious problem - resolve or explain
   0 ALERT level B = A potentially serious problem, consider carefully
   1 ALERT level C = Check. Ensure it is not caused by an omission or oversight
   2 ALERT level G = General information/check it is not something unexpected

   1 ALERT type 1 CIF construction/syntax error, inconsistent or missing data
   0 ALERT type 2 Indicator that the structure model may be wrong or deficient
   1 ALERT type 3 Indicator that the structure quality may be low
   1 ALERT type 4 Improvement, methodology, query or suggestion
   0 ALERT type 5 Informative message, check
```

It is advisable to attempt to resolve as many as possible of the alerts in all categories. Often the minor alerts point to easily fixed oversights, errors and omissions in your CIF or refinement strategy, so attention to these fine details can be worthwhile. In order to resolve some of the more serious problems it may be necessary to carry out additional measurements or structure refinements. However, the purpose of your study may justify the reported deviations and the more serious of these should normally be commented upon in the discussion or experimental section of a paper or in the "special_details" fields of the CIF. checkCIF was carefully designed to identify outliers and unusual parameters, but every test has its limitations and alerts that are not important in a particular case may appear. Conversely, the absence of alerts does not guarantee there are no aspects of the results needing attention. It is up to the individual to critically assess their own results and, if necessary, seek expert advice.

Publication of your CIF in IUCr journals

A basic structural check has been run on your CIF. These basic checks will be run on all CIFs submitted for publication in IUCr journals (*Acta Crystallographica*, *Journal of Applied Crystallography*, *Journal of Synchrotron Radiation*); however, if you intend to submit to *Acta Crystallographica Section C* or *E*, you should make sure that full publication checks are run on the final version of your CIF prior to submission.

Publication of your CIF in other journals

Please refer to the *Notes for Authors* of the relevant journal for any special instructions relating to CIF submission.

PLATON version of 24/07/2014; check.def file version of 24/07/2014

Datablock mo_ph3_0m - ellipsoid plot

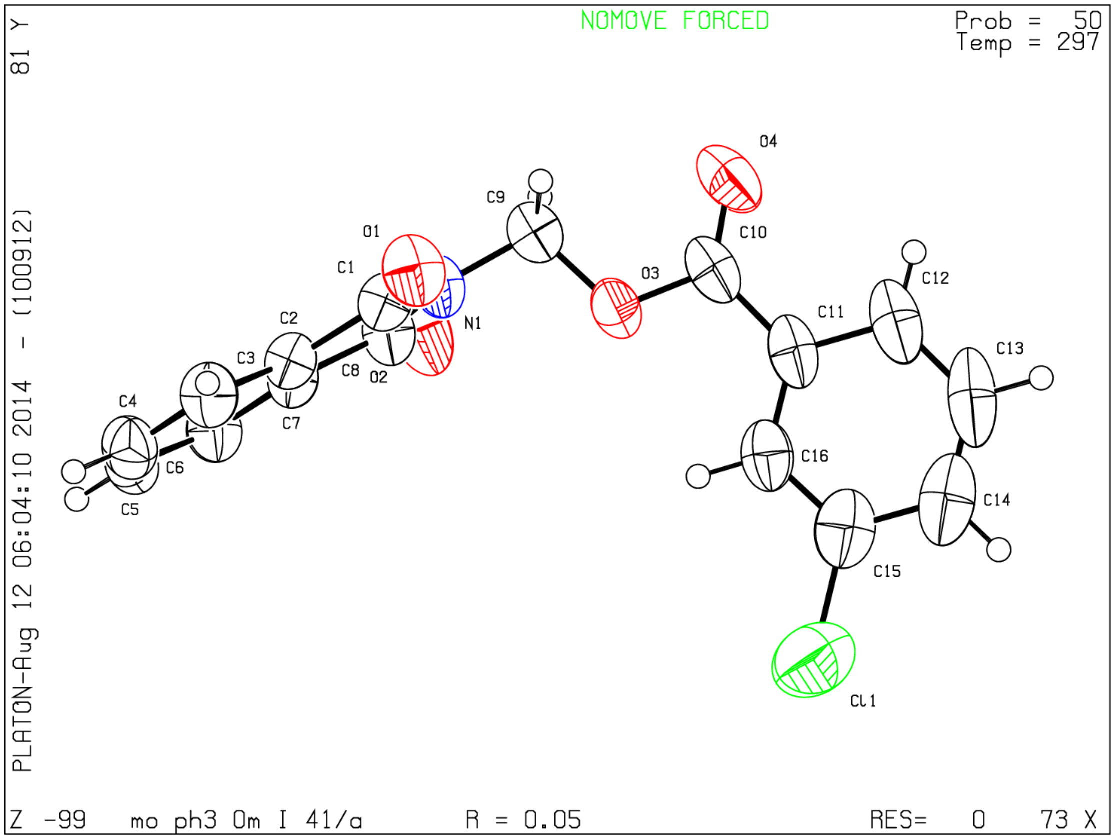

checkCIF/PLATON report

Structure factors have been supplied for datablock(s) mo_ph5_0m

THIS REPORT IS FOR GUIDANCE ONLY. IF USED AS PART OF A REVIEW PROCEDURE FOR PUBLICATION, IT SHOULD NOT REPLACE THE EXPERTISE OF AN EXPERIENCED CRYSTALLOGRAPHIC REFEREE.

No syntax errors found.   CIF dictionary   Interpreting this report

Datablock: mo_ph5_0m

Bond precision: C-C = 0.0043 A   Wavelength=0.71073

Cell:	a=14.340(3)	b=13.951(2)	c=7.6331(13)
	alpha=90	beta=103.005(3)	gamma=90
Temperature:	297 K		

	Calculated	Reported
Volume	1487.9(5)	1487.9(4)
Space group	P 21/c	P 21/c
Hall group	-P 2ybc	-P 2ybc
Moiety formula	C16 H9 Cl2 N O4	C16 H9 Cl2 N O4
Sum formula	C16 H9 Cl2 N O4	C16 H9 Cl2 N O4
Mr	350.14	350.14
Dx,g cm-3	1.563	1.563
Z	4	4
Mu (mm-1)	0.456	0.456
F000	712.0	712.0
F000’	713.51	
h,k,lmax	19,19,10	19,19,10
Nref	3994	3964
Tmin,Tmax	0.901,0.960	0.781,0.962
Tmin’	0.771	

Correction method= MULTI-SCAN

Data completeness= 0.992   Theta(max)= 29.090

R(reflections)= 0.0488(1900)   wR2(reflections)= 0.1994(3964)

S = 0.993   Npar= 257

The following ALERTS were generated. Each ALERT has the format
test-name_ALERT_alert-type_alert-level.
Click on the hyperlinks for more details of the test.

Alert level C

```
PLAT026_ALERT_3_C Ratio Observed / Unique Reflections too Low ....         48 %
PLAT213_ALERT_2_C Atom C11B            has ADP max/min Ratio .....        3.4 prolat
PLAT230_ALERT_2_C Hirshfeld Test Diff for    C1      --  C2      ..        7.0 su
PLAT234_ALERT_4_C Large Hirshfeld Difference C10     --  C11B    ..       0.22 Ang.
PLAT340_ALERT_3_C Low Bond Precision on  C-C Bonds ..............     0.0043 Ang.
PLAT906_ALERT_3_C Large K value in the Analysis of Variance ......      5.531 Check
PLAT911_ALERT_3_C Missing # FCF Refl Between THmin & STh/L=  0.600          7 Report
```

Alert level G

```
PLAT072_ALERT_2_G SHELXL First  Parameter in WGHT Unusually Large.       0.11 Report
PLAT171_ALERT_4_G The CIF-Embedded .res File Contains EADP Records          2 Report
PLAT301_ALERT_3_G Main Residue  Disorder ............ Percentage =         35 Note
PLAT779_ALERT_4_G Suspect or Irrelevant (Bond) Angle in CIF .... #         40 Check
              C11B -C10  -C11A    1.555   1.555   1.555              8.10 Deg.
PLAT811_ALERT_5_G No ADDSYM Analysis: Too Many Excluded Atoms ....          ! Info
PLAT912_ALERT_4_G Missing # of FCF Reflections Above STh/L=  0.600         24 Note
```

---

0 **ALERT level A** = Most likely a serious problem - resolve or explain
0 **ALERT level B** = A potentially serious problem, consider carefully
7 **ALERT level C** = Check. Ensure it is not caused by an omission or oversight
6 **ALERT level G** = General information/check it is not something unexpected

0 ALERT type 1 CIF construction/syntax error, inconsistent or missing data
3 ALERT type 2 Indicator that the structure model may be wrong or deficient
5 ALERT type 3 Indicator that the structure quality may be low
4 ALERT type 4 Improvement, methodology, query or suggestion
1 ALERT type 5 Informative message, check

It is advisable to attempt to resolve as many as possible of the alerts in all categories. Often the minor alerts point to easily fixed oversights, errors and omissions in your CIF or refinement strategy, so attention to these fine details can be worthwhile. In order to resolve some of the more serious problems it may be necessary to carry out additional measurements or structure refinements. However, the purpose of your study may justify the reported deviations and the more serious of these should normally be commented upon in the discussion or experimental section of a paper or in the "special_details" fields of the CIF. checkCIF was carefully designed to identify outliers and unusual parameters, but every test has its limitations and alerts that are not important in a particular case may appear. Conversely, the absence of alerts does not guarantee there are no aspects of the results needing attention. It is up to the individual to critically assess their own results and, if necessary, seek expert advice.

Publication of your CIF in IUCr journals

A basic structural check has been run on your CIF. These basic checks will be run on all CIFs submitted for publication in IUCr journals (*Acta Crystallographica*, *Journal of Applied Crystallography*, *Journal of Synchrotron Radiation*); however, if you intend to submit to *Acta Crystallographica Section C* or *E*, you should make sure that full publication checks are run on the final version of your CIF prior to submission.

Publication of your CIF in other journals

Please refer to the *Notes for Authors* of the relevant journal for any special instructions relating to CIF submission.

PLATON version of 24/07/2014; check.def file version of 24/07/2014

Datablock mo_ph5_0m - ellipsoid plot

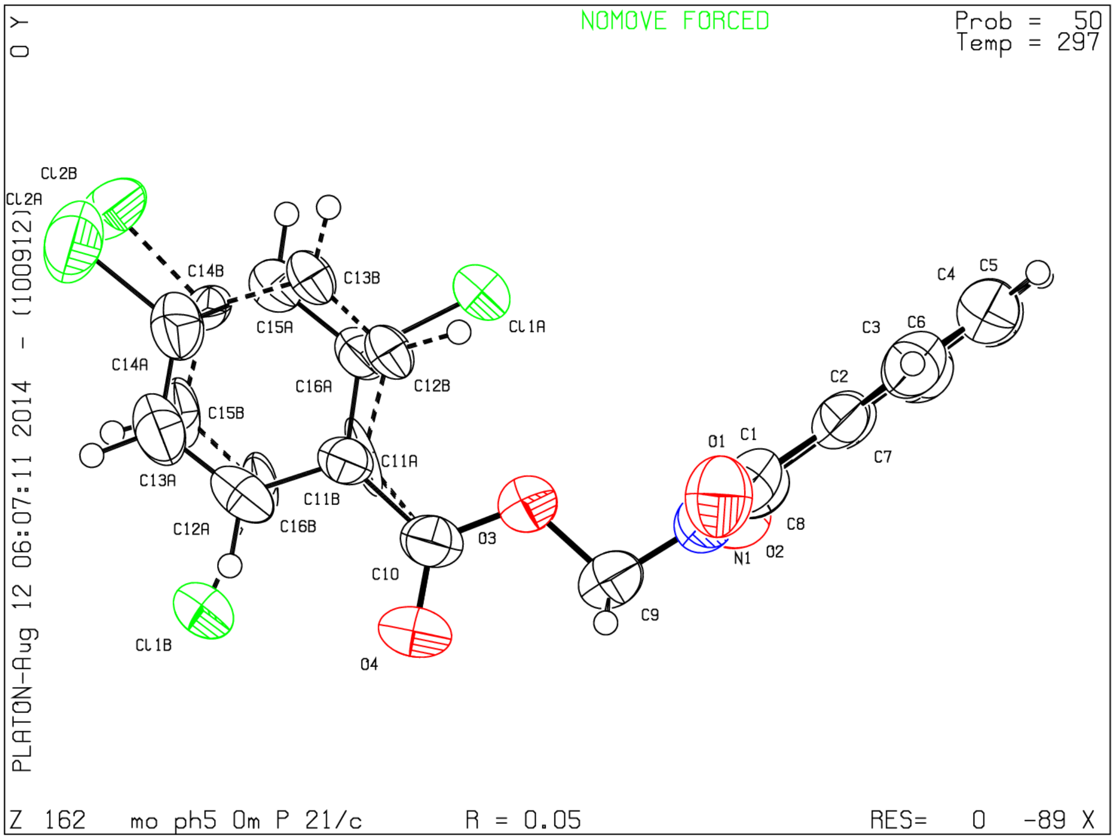

checkCIF/PLATON report

Structure factors have been supplied for datablock(s) mo_ph6_0m

THIS REPORT IS FOR GUIDANCE ONLY. IF USED AS PART OF A REVIEW PROCEDURE FOR PUBLICATION, IT SHOULD NOT REPLACE THE EXPERTISE OF AN EXPERIENCED CRYSTALLOGRAPHIC REFEREE.

No syntax errors found. CIF dictionary Interpreting this report

Datablock: mo_ph6_0m

```
Bond precision:  C-C = 0.0018 A          Wavelength=0.71073

Cell:          a=7.3517(5)       b=7.6033(5)      c=13.6578(10)
               alpha=98.867(2)   beta=99.506(3)   gamma=110.9794(14)
Temperature:   100 K

                 Calculated                 Reported
Volume           683.92(8)                  683.93(8)
Space group      P -1                       P -1
Hall group       -P 1                       -P 1
Moiety formula   C17 H13 N O4               C17 H13 N O4
Sum formula      C17 H13 N O4               C17 H13 N O4
Mr               295.28                     295.28
Dx,g cm-3        1.434                      1.434
Z                2                          2
Mu (mm-1)        0.103                      0.103
F000             308.0                      308.0
F000'            308.17
h,k,lmax         10,10,19                   10,10,19
Nref             4199                       4183
Tmin,Tmax        0.952,0.961                0.946,0.961
Tmin'            0.946

Correction method= MULTI-SCAN

Data completeness= 0.996          Theta(max)= 30.580

R(reflections)= 0.0502( 3359)     wR2(reflections)= 0.1405( 4183)

S = 1.065                  Npar= 200
```

The following ALERTS were generated. Each ALERT has the format
test-name_ALERT_alert-type_alert-level.
Click on the hyperlinks for more details of the test.

Alert level C

```
PLAT906_ALERT_3_C Large K value in the Analysis of Variance ......      2.179 Check
```

Alert level G

```
PLAT066_ALERT_1_G Predicted and Reported Tmin&Tmax Range Identical          ? Check
PLAT910_ALERT_3_G Missing # of FCF Reflections Below Th(Min) .....          1 Report
PLAT912_ALERT_4_G Missing # of FCF Reflections Above STh/L=  0.600         15 Note
```

```
  0 ALERT level A = Most likely a serious problem - resolve or explain
  0 ALERT level B = A potentially serious problem, consider carefully
  1 ALERT level C = Check. Ensure it is not caused by an omission or oversight
  3 ALERT level G = General information/check it is not something unexpected

  1 ALERT type 1 CIF construction/syntax error, inconsistent or missing data
  0 ALERT type 2 Indicator that the structure model may be wrong or deficient
  2 ALERT type 3 Indicator that the structure quality may be low
  1 ALERT type 4 Improvement, methodology, query or suggestion
  0 ALERT type 5 Informative message, check
```

It is advisable to attempt to resolve as many as possible of the alerts in all categories. Often the minor alerts point to easily fixed oversights, errors and omissions in your CIF or refinement strategy, so attention to these fine details can be worthwhile. In order to resolve some of the more serious problems it may be necessary to carry out additional measurements or structure refinements. However, the purpose of your study may justify the reported deviations and the more serious of these should normally be commented upon in the discussion or experimental section of a paper or in the "special_details" fields of the CIF. checkCIF was carefully designed to identify outliers and unusual parameters, but every test has its limitations and alerts that are not important in a particular case may appear. Conversely, the absence of alerts does not guarantee there are no aspects of the results needing attention. It is up to the individual to critically assess their own results and, if necessary, seek expert advice.

Publication of your CIF in IUCr journals

A basic structural check has been run on your CIF. These basic checks will be run on all CIFs submitted for publication in IUCr journals (*Acta Crystallographica*, *Journal of Applied Crystallography*, *Journal of Synchrotron Radiation*); however, if you intend to submit to *Acta Crystallographica Section C* or *E*, you should make sure that full publication checks are run on the final version of your CIF prior to submission.

Publication of your CIF in other journals

Please refer to the *Notes for Authors* of the relevant journal for any special instructions relating to CIF submission.

PLATON version of 24/07/2014; check.def file version of 24/07/2014

Datablock mo_ph6_0m - ellipsoid plot

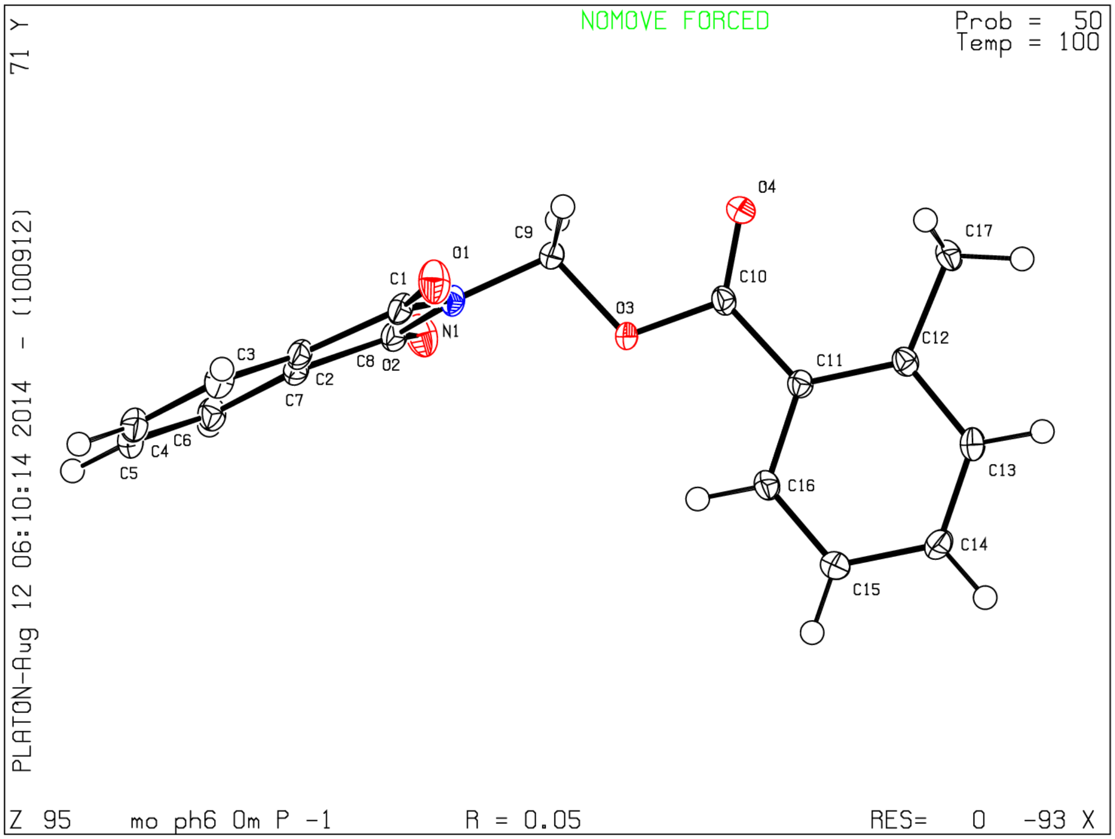

checkCIF/PLATON report

Structure factors have been supplied for datablock(s) mo_ph7_0m

THIS REPORT IS FOR GUIDANCE ONLY. IF USED AS PART OF A REVIEW PROCEDURE FOR PUBLICATION, IT SHOULD NOT REPLACE THE EXPERTISE OF AN EXPERIENCED CRYSTALLOGRAPHIC REFEREE.

No syntax errors found.   CIF dictionary   Interpreting this report

Datablock: mo_ph7_0m

```
Bond precision:  C-C = 0.0030 A          Wavelength=0.71073

Cell:            a=4.3984(2)     b=13.9960(8)       c=22.4870(12)
                 alpha=90        beta=97.622(2)     gamma=90
Temperature:     100 K

                Calculated                Reported
Volume          1372.07(12)               1372.07(12)
Space group     P 21/c                    P 21/c
Hall group      -P 2ybc                   -P 2ybc
Moiety formula  C17 H13 N O4              C17 H13 N O4
Sum formula     C17 H13 N O4              C17 H13 N O4
Mr              295.28                    295.28
Dx,g cm-3       1.429                     1.429
Z               4                         4
Mu (mm-1)       0.103                     0.103
F000            616.0                     616.0
F000'           616.33
h,k,lmax        6,20,32                   6,20,32
Nref            4240                      4235
Tmin,Tmax       0.991,0.994               0.931,0.994
Tmin'           0.930

Correction method= MULTI-SCAN

Data completeness= 0.999          Theta(max)= 30.630

R(reflections)= 0.0608( 2400)     wR2(reflections)= 0.1303( 4235)

S = 1.005                  Npar= 200
```

The following ALERTS were generated. Each ALERT has the format
test-name_ALERT_alert-type_alert-level.
Click on the hyperlinks for more details of the test.

Alert level C

```
RINTA01_ALERT_3_C  The value of Rint is greater than 0.12
            Rint given   0.158
PLAT906_ALERT_3_C Large K value in the Analysis of Variance ......      3.452 Check
```

Alert level G

```
PLAT063_ALERT_4_G Crystal Size Likely too Large for Beam Size ....       0.70 mm
PLAT128_ALERT_4_G Alternate Setting for Input Space Group  P21/c       P21/n Note
PLAT910_ALERT_3_G Missing # of FCF Reflections Below Th(Min) .....          2 Report
PLAT912_ALERT_4_G Missing # of FCF Reflections Above STh/L=  0.600          3 Note
```

```
   0 ALERT level A = Most likely a serious problem - resolve or explain
   0 ALERT level B = A potentially serious problem, consider carefully
   2 ALERT level C = Check. Ensure it is not caused by an omission or oversight
   4 ALERT level G = General information/check it is not something unexpected

   0 ALERT type 1 CIF construction/syntax error, inconsistent or missing data
   0 ALERT type 2 Indicator that the structure model may be wrong or deficient
   3 ALERT type 3 Indicator that the structure quality may be low
   3 ALERT type 4 Improvement, methodology, query or suggestion
   0 ALERT type 5 Informative message, check
```

It is advisable to attempt to resolve as many as possible of the alerts in all categories. Often the minor alerts point to easily fixed oversights, errors and omissions in your CIF or refinement strategy, so attention to these fine details can be worthwhile. In order to resolve some of the more serious problems it may be necessary to carry out additional measurements or structure refinements. However, the purpose of your study may justify the reported deviations and the more serious of these should normally be commented upon in the discussion or experimental section of a paper or in the "special_details" fields of the CIF. checkCIF was carefully designed to identify outliers and unusual parameters, but every test has its limitations and alerts that are not important in a particular case may appear. Conversely, the absence of alerts does not guarantee there are no aspects of the results needing attention. It is up to the individual to critically assess their own results and, if necessary, seek expert advice.

Publication of your CIF in IUCr journals

A basic structural check has been run on your CIF. These basic checks will be run on all CIFs submitted for publication in IUCr journals (*Acta Crystallographica*, *Journal of Applied Crystallography*, *Journal of Synchrotron Radiation*); however, if you intend to submit to *Acta Crystallographica Section C* or *E*, you should make sure that full publication checks are run on the final version of your CIF prior to submission.

Publication of your CIF in other journals

Please refer to the *Notes for Authors* of the relevant journal for any special instructions relating to CIF submission.

PLATON version of 24/07/2014; check.def file version of 24/07/2014

Datablock mo_ph7_0m - ellipsoid plot

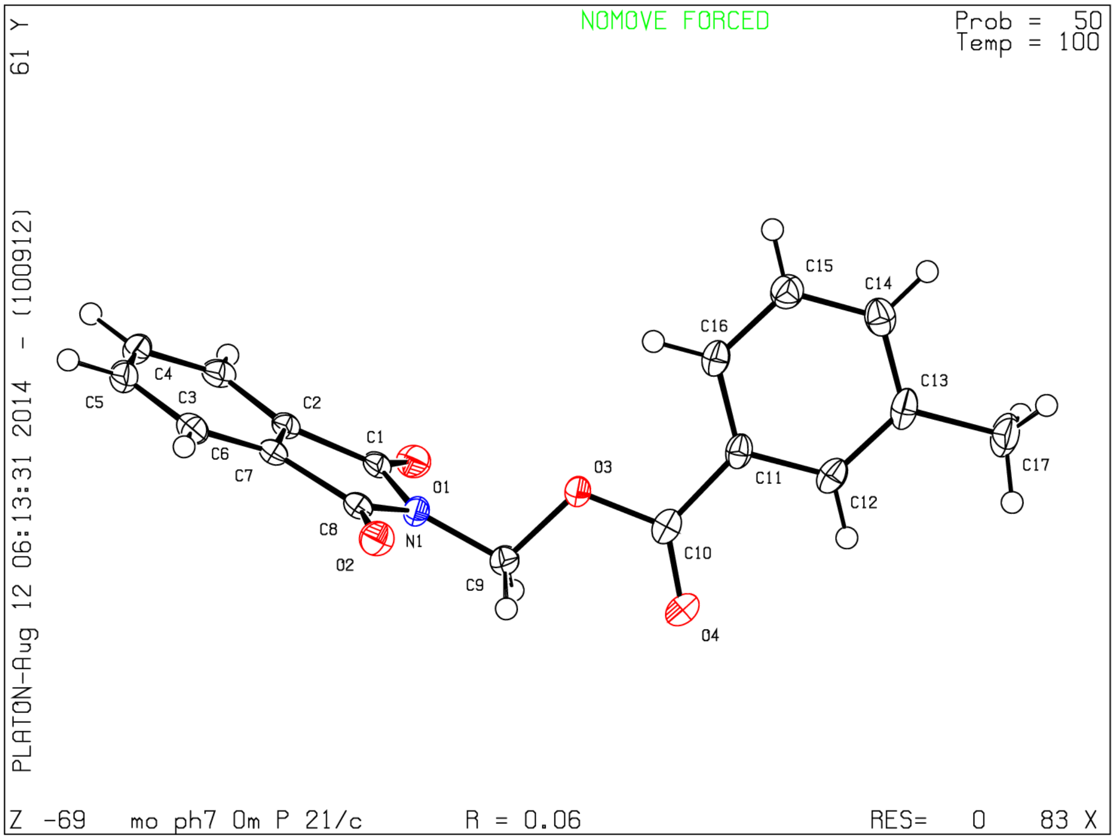

checkCIF/PLATON report

Structure factors have been supplied for datablock(s) mo_ph9_0m

THIS REPORT IS FOR GUIDANCE ONLY. IF USED AS PART OF A REVIEW PROCEDURE FOR PUBLICATION, IT SHOULD NOT REPLACE THE EXPERTISE OF AN EXPERIENCED CRYSTALLOGRAPHIC REFEREE.

No syntax errors found.   CIF dictionary   Interpreting this report

Datablock: mo_ph9_0m

```
Bond precision:  C-C = 0.0023 A          Wavelength=0.71073

Cell:             a=12.4243(8)      b=7.1597(5)      c=32.917(2)
                  alpha=90          beta=90          gamma=90
Temperature:      297 K

                Calculated                  Reported
Volume          2928.1(3)                   2928.1(3)
Space group     P b c a                     P b c a
Hall group      -P 2ac 2ab                  -P 2ac 2ab
Moiety formula  C17 H13 N O5                C17 H13 N O5
Sum formula     C17 H13 N O5                C17 H13 N O5
Mr              311.28                      311.28
Dx,g cm-3       1.412                       1.412
Z               8                           8
Mu (mm-1)       0.105                       0.105
F000            1296.0                      1296.0
F000'           1296.74
h,k,lmax        17,10,46                    17,10,46
Nref            4426                        4405
Tmin,Tmax       0.947,0.981                 0.947,0.981
Tmin'           0.947

Correction method= MULTI-SCAN

Data completeness= 0.995          Theta(max)= 30.390

R(reflections)= 0.0448( 3155)     wR2(reflections)= 0.1378( 4405)

S = 1.034                  Npar= 209
```

The following ALERTS were generated. Each ALERT has the format
test-name_ALERT_alert-type_alert-level.
Click on the hyperlinks for more details of the test.

Alert level C

```
PLAT230_ALERT_2_C Hirshfeld Test Diff for    C13    --  C14     ..        5.5 su
```

Alert level G

```
PLAT066_ALERT_1_G Predicted and Reported Tmin&Tmax Range Identical          ? Check
PLAT912_ALERT_4_G Missing # of FCF Reflections Above STh/L=  0.600        21 Note
```

```
   0 ALERT level A = Most likely a serious problem - resolve or explain
   0 ALERT level B = A potentially serious problem, consider carefully
   1 ALERT level C = Check. Ensure it is not caused by an omission or oversight
   2 ALERT level G = General information/check it is not something unexpected

   1 ALERT type 1 CIF construction/syntax error, inconsistent or missing data
   1 ALERT type 2 Indicator that the structure model may be wrong or deficient
   0 ALERT type 3 Indicator that the structure quality may be low
   1 ALERT type 4 Improvement, methodology, query or suggestion
   0 ALERT type 5 Informative message, check
```

It is advisable to attempt to resolve as many as possible of the alerts in all categories. Often the minor alerts point to easily fixed oversights, errors and omissions in your CIF or refinement strategy, so attention to these fine details can be worthwhile. In order to resolve some of the more serious problems it may be necessary to carry out additional measurements or structure refinements. However, the purpose of your study may justify the reported deviations and the more serious of these should normally be commented upon in the discussion or experimental section of a paper or in the "special_details" fields of the CIF. checkCIF was carefully designed to identify outliers and unusual parameters, but every test has its limitations and alerts that are not important in a particular case may appear. Conversely, the absence of alerts does not guarantee there are no aspects of the results needing attention. It is up to the individual to critically assess their own results and, if necessary, seek expert advice.

Publication of your CIF in IUCr journals

A basic structural check has been run on your CIF. These basic checks will be run on all CIFs submitted for publication in IUCr journals (*Acta Crystallographica*, *Journal of Applied Crystallography*, *Journal of Synchrotron Radiation*); however, if you intend to submit to *Acta Crystallographica Section C* or *E*, you should make sure that full publication checks are run on the final version of your CIF prior to submission.

Publication of your CIF in other journals

Please refer to the *Notes for Authors* of the relevant journal for any special instructions relating to CIF submission.

PLATON version of 24/07/2014; check.def file version of 24/07/2014

Datablock mo_ph9_0m - ellipsoid plot

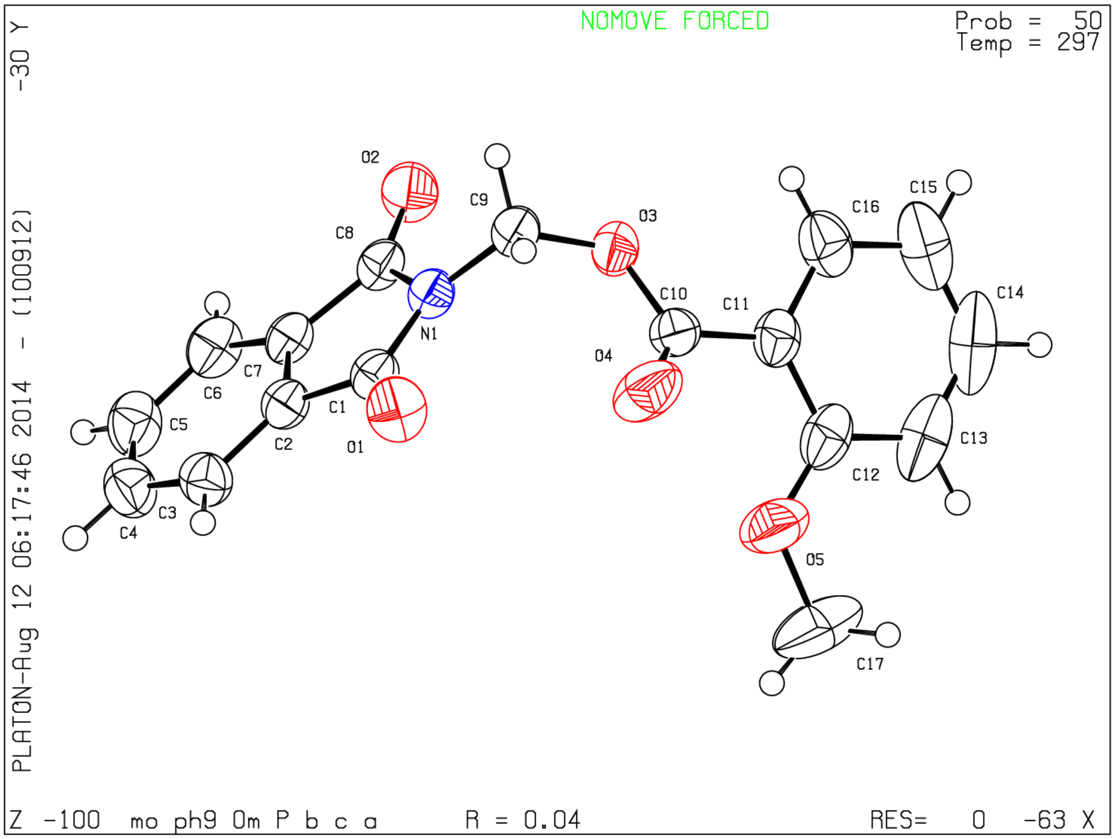

checkCIF/PLATON report

Structure factors have been supplied for datablock(s) mo_ph10_0m

THIS REPORT IS FOR GUIDANCE ONLY. IF USED AS PART OF A REVIEW PROCEDURE FOR PUBLICATION, IT SHOULD NOT REPLACE THE EXPERTISE OF AN EXPERIENCED CRYSTALLOGRAPHIC REFEREE.

No syntax errors found. CIF dictionary Interpreting this report

Datablock: mo_ph10_0m

```
Bond precision:  C-C = 0.0021 A          Wavelength=0.71073

Cell:          a=8.4216(5)      b=8.4471(6)       c=10.8739(7)
               alpha=80.311(2)  beta=86.3815(18)  gamma=66.3040(19)
Temperature:   100 K
```

	Calculated	Reported
Volume	698.22(8)	698.22(8)
Space group	P -1	P -1
Hall group	-P 1	-P 1
Moiety formula	C17 H13 N O5	C17 H13 N O5
Sum formula	C17 H13 N O5	C17 H13 N O5
Mr	311.28	311.28
Dx,g cm-3	1.481	1.481
Z	2	2
Mu (mm-1)	0.110	0.110
F000	324.0	324.0
F000’	324.19	
h,k,lmax	12,12,15	12,12,15
Nref	4302	4282
Tmin,Tmax	0.951,0.983	0.951,0.983
Tmin’	0.951	

```
Correction method= MULTI-SCAN

Data completeness= 0.995          Theta(max)= 30.610

R(reflections)= 0.0678( 3198)     wR2(reflections)= 0.1928( 4282)

S = 1.022                  Npar= 209
```

The following ALERTS were generated. Each ALERT has the format
test-name_ALERT_alert-type_alert-level.
Click on the hyperlinks for more details of the test.

Alert level C

```
PLAT213_ALERT_2_C Atom C1              has ADP max/min Ratio .....        3.1 oblate
PLAT213_ALERT_2_C Atom C9              has ADP max/min Ratio .....        3.2 oblate
PLAT250_ALERT_2_C Large U3/U1 Ratio for Average U(i,j) Tensor ....        2.6 Note
PLAT906_ALERT_3_C Large K value in the Analysis of Variance ......      3.248 Check
PLAT911_ALERT_3_C Missing # FCF Refl Between THmin & STh/L=  0.600          2 Report
PLAT975_ALERT_2_C Check Calcd Residual Density  0.87A From      O2       0.51 eA-3
PLAT975_ALERT_2_C Check Calcd Residual Density  0.94A From      O1       0.46 eA-3
```

Alert level G

```
PLAT066_ALERT_1_G Predicted and Reported Tmin&Tmax Range Identical          ? Check
PLAT072_ALERT_2_G SHELXL First  Parameter in WGHT Unusually Large.       0.13 Report
PLAT432_ALERT_2_G Short Inter X...Y Contact  O5     ..  C8      ..       3.00 Ang.
PLAT910_ALERT_3_G Missing # of FCF Reflections Below Th(Min) .....          1 Report
PLAT912_ALERT_4_G Missing # of FCF Reflections Above STh/L=  0.600         18 Note
```

```
   0 ALERT level A = Most likely a serious problem - resolve or explain
   0 ALERT level B = A potentially serious problem, consider carefully
   7 ALERT level C = Check. Ensure it is not caused by an omission or oversight
   5 ALERT level G = General information/check it is not something unexpected

   1 ALERT type 1 CIF construction/syntax error, inconsistent or missing data
   7 ALERT type 2 Indicator that the structure model may be wrong or deficient
   3 ALERT type 3 Indicator that the structure quality may be low
   1 ALERT type 4 Improvement, methodology, query or suggestion
   0 ALERT type 5 Informative message, check
```

It is advisable to attempt to resolve as many as possible of the alerts in all categories. Often the minor alerts point to easily fixed oversights, errors and omissions in your CIF or refinement strategy, so attention to these fine details can be worthwhile. In order to resolve some of the more serious problems it may be necessary to carry out additional measurements or structure refinements. However, the purpose of your study may justify the reported deviations and the more serious of these should normally be commented upon in the discussion or experimental section of a paper or in the "special_details" fields of the CIF. checkCIF was carefully designed to identify outliers and unusual parameters, but every test has its limitations and alerts that are not important in a particular case may appear. Conversely, the absence of alerts does not guarantee there are no aspects of the results needing attention. It is up to the individual to critically assess their own results and, if necessary, seek expert advice.

Publication of your CIF in IUCr journals

A basic structural check has been run on your CIF. These basic checks will be run on all CIFs submitted for publication in IUCr journals (*Acta Crystallographica*, *Journal of Applied Crystallography*, *Journal of Synchrotron Radiation*); however, if you intend to submit to *Acta Crystallographica Section C* or *E*, you should make sure that full publication checks are run on the final version of your CIF prior to submission.

Publication of your CIF in other journals

Please refer to the *Notes for Authors* of the relevant journal for any special instructions relating to CIF submission.

PLATON version of 24/07/2014; check.def file version of 24/07/2014

Datablock mo_ph10_0m - ellipsoid plot

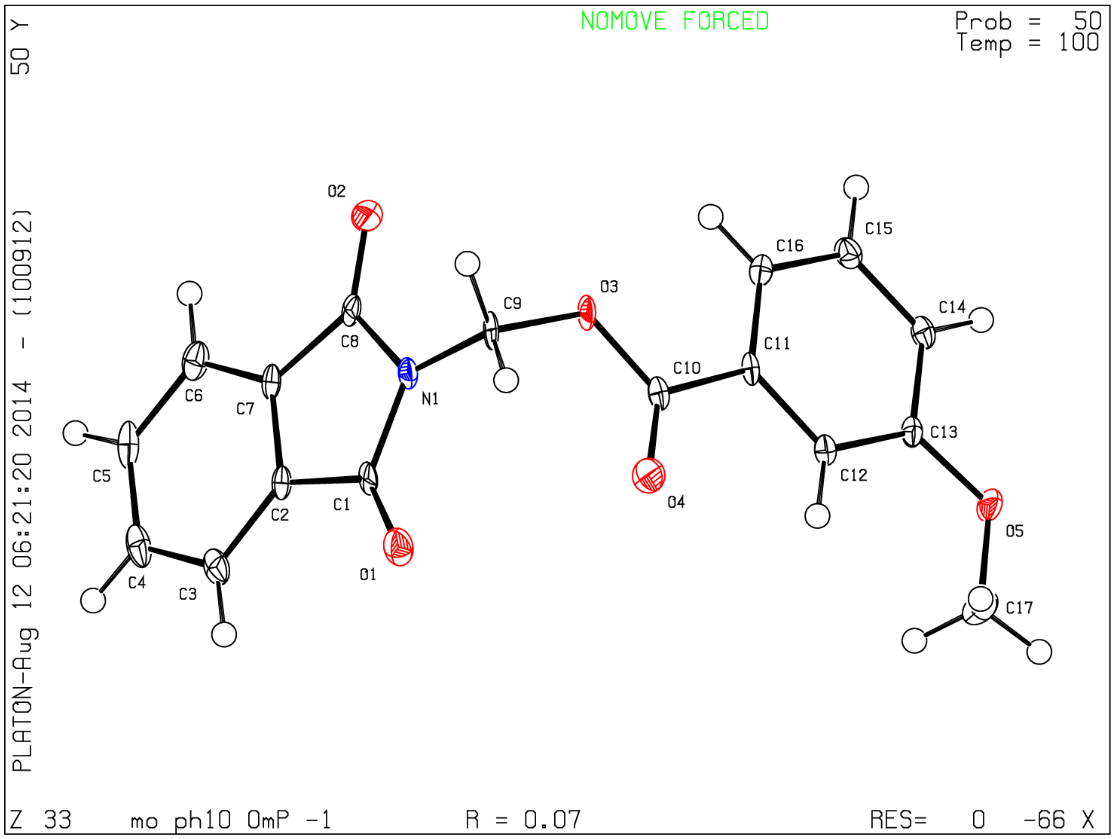

checkCIF/PLATON report

Structure factors have been supplied for datablock(s) mo_ph11_0m

THIS REPORT IS FOR GUIDANCE ONLY. IF USED AS PART OF A REVIEW PROCEDURE FOR PUBLICATION, IT SHOULD NOT REPLACE THE EXPERTISE OF AN EXPERIENCED CRYSTALLOGRAPHIC REFEREE.

No syntax errors found. CIF dictionary Interpreting this report

Datablock: mo_ph11_0m

Bond precision: C-C = 0.0029 A Wavelength=0.71073

Cell:	a=14.2125(8)	b=13.0603(8)	c=7.9266(4)
	alpha=90	beta=100.672(2)	gamma=90
Temperature:	100 K		

	Calculated	Reported
Volume	1445.88(14)	1445.88(14)
Space group	P 21/c	P 21/c
Hall group	-P 2ybc	-P 2ybc
Moiety formula	C17 H13 N O5	C17 H13 N O5
Sum formula	C17 H13 N O5	C17 H13 N O5
Mr	311.28	311.28
Dx,g cm-3	1.430	1.430
Z	4	4
Mu (mm-1)	0.107	0.107
F000	648.0	648.0
F000’	648.37	
h,k,lmax	20,18,11	20,18,11
Nref	4456	4446
Tmin,Tmax	0.956,0.988	0.942,0.988
Tmin’	0.941	

Correction method= MULTI-SCAN

Data completeness= 0.998 Theta(max)= 30.610

R(reflections)= 0.0747(2696) wR2(reflections)= 0.2105(4446)

S = 1.001 Npar= 209

The following ALERTS were generated. Each ALERT has the format
test-name_ALERT_alert-type_alert-level.
Click on the hyperlinks for more details of the test.

Alert level C

```
RINTA01_ALERT_3_C  The value of Rint is greater than 0.12
            Rint given   0.167
PLAT020_ALERT_3_C The value of Rint is greater than 0.12 .........      0.167 Report
PLAT905_ALERT_3_C Negative K value in the Analysis of Variance ...     -6.040 Report
PLAT975_ALERT_2_C Check Calcd Residual Density  0.80A From      O2       0.46 eA-3
```

Alert level G

```
PLAT072_ALERT_2_G SHELXL First  Parameter in WGHT Unusually Large.       0.12 Report
PLAT910_ALERT_3_G Missing # of FCF Reflections Below Th(Min) .....          2 Report
PLAT912_ALERT_4_G Missing # of FCF Reflections Above STh/L=  0.600          9 Note
```

```
   0 ALERT level A = Most likely a serious problem - resolve or explain
   0 ALERT level B = A potentially serious problem, consider carefully
   4 ALERT level C = Check. Ensure it is not caused by an omission or oversight
   3 ALERT level G = General information/check it is not something unexpected

   0 ALERT type 1 CIF construction/syntax error, inconsistent or missing data
   2 ALERT type 2 Indicator that the structure model may be wrong or deficient
   4 ALERT type 3 Indicator that the structure quality may be low
   1 ALERT type 4 Improvement, methodology, query or suggestion
   0 ALERT type 5 Informative message, check
```

It is advisable to attempt to resolve as many as possible of the alerts in all categories. Often the minor alerts point to easily fixed oversights, errors and omissions in your CIF or refinement strategy, so attention to these fine details can be worthwhile. In order to resolve some of the more serious problems it may be necessary to carry out additional measurements or structure refinements. However, the purpose of your study may justify the reported deviations and the more serious of these should normally be commented upon in the discussion or experimental section of a paper or in the "special_details" fields of the CIF. checkCIF was carefully designed to identify outliers and unusual parameters, but every test has its limitations and alerts that are not important in a particular case may appear. Conversely, the absence of alerts does not guarantee there are no aspects of the results needing attention. It is up to the individual to critically assess their own results and, if necessary, seek expert advice.

Publication of your CIF in IUCr journals

A basic structural check has been run on your CIF. These basic checks will be run on all CIFs submitted for publication in IUCr journals (*Acta Crystallographica*, *Journal of Applied Crystallography*, *Journal of Synchrotron Radiation*); however, if you intend to submit to *Acta Crystallographica Section C* or *E*, you should make sure that full publication checks are run on the final version of your CIF prior to submission.

Publication of your CIF in other journals

Please refer to the *Notes for Authors* of the relevant journal for any special instructions relating to CIF submission.

PLATON version of 24/07/2014; check.def file version of 24/07/2014

Datablock mo_ph11_0m - ellipsoid plot

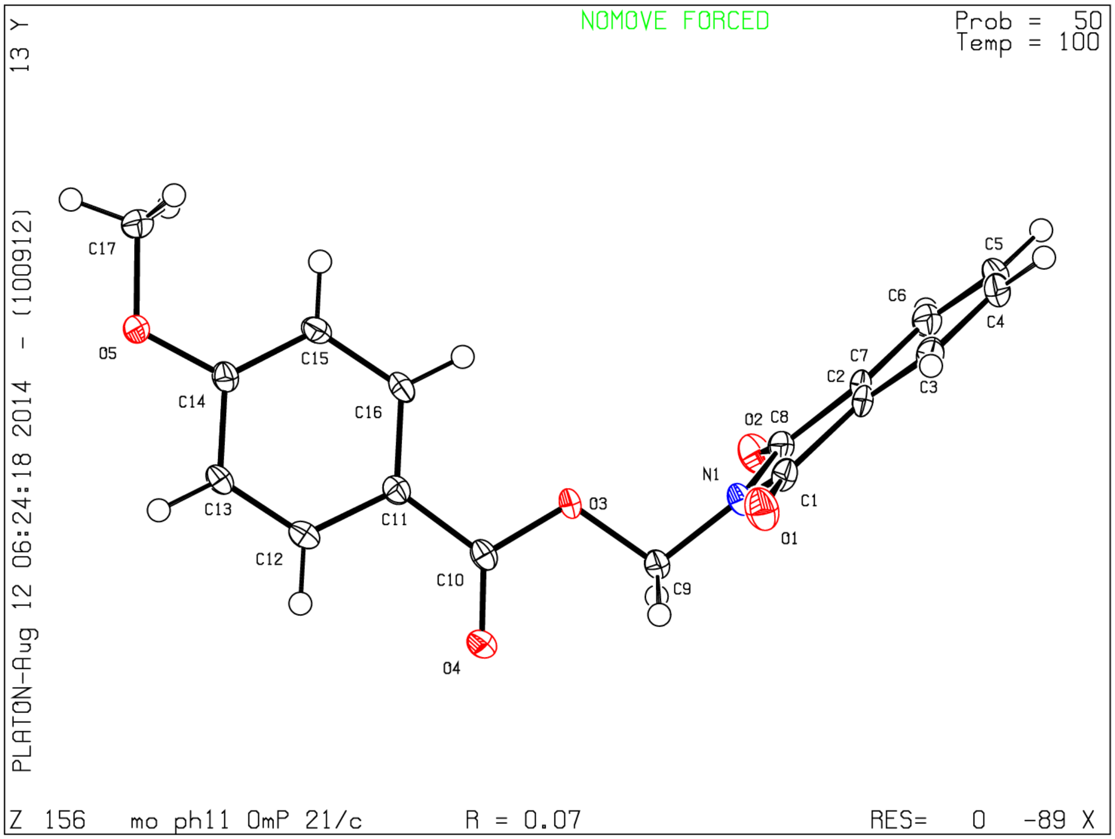

checkCIF/PLATON report

Structure factors have been supplied for datablock(s) mo_ph15_0m

THIS REPORT IS FOR GUIDANCE ONLY. IF USED AS PART OF A REVIEW PROCEDURE FOR PUBLICATION, IT SHOULD NOT REPLACE THE EXPERTISE OF AN EXPERIENCED CRYSTALLOGRAPHIC REFEREE.

No syntax errors found.   CIF dictionary   Interpreting this report

Datablock: mo_ph15_0m

```
Bond precision:  C-C = 0.0019 A          Wavelength=0.71073

Cell:         a=7.5556(5)       b=7.6118(4)      c=13.6551(8)
              alpha=99.1319(17) beta=97.0675(19) gamma=113.2039(16)
Temperature: 100 K

                Calculated                  Reported
Volume          697.59(7)                   697.59(7)
Space group     P -1                        P -1
Hall group      -P 1                        -P 1
Moiety formula  C16 H10 N2 O6               C16 H10 N2 O6
Sum formula     C16 H10 N2 O6               C16 H10 N2 O6
Mr              326.26                      326.26
Dx,g cm-3       1.553                       1.553
Z               2                           2
Mu (mm-1)       0.122                       0.122
F000            336.0                       336.0
F000'           336.20
h,k,lmax        10,10,19                    10,10,19
Nref            4281                        4279
Tmin,Tmax       0.964,0.981                 0.935,0.981
Tmin'           0.934

Correction method= MULTI-SCAN

Data completeness= 1.000          Theta(max)= 30.600

R(reflections)= 0.0472( 3327)     wR2(reflections)= 0.1297( 4279)

S = 1.038                   Npar= 217
```

The following ALERTS were generated. Each ALERT has the format
test-name_ALERT_alert-type_alert-level.
Click on the hyperlinks for more details of the test.

Alert level G

```
PLAT910_ALERT_3_G Missing # of FCF Reflections Below Th(Min) .....         1 Report
```

```
   0 ALERT level A = Most likely a serious problem - resolve or explain
   0 ALERT level B = A potentially serious problem, consider carefully
   0 ALERT level C = Check. Ensure it is not caused by an omission or oversight
   1 ALERT level G = General information/check it is not something unexpected

   0 ALERT type 1 CIF construction/syntax error, inconsistent or missing data
   0 ALERT type 2 Indicator that the structure model may be wrong or deficient
   1 ALERT type 3 Indicator that the structure quality may be low
   0 ALERT type 4 Improvement, methodology, query or suggestion
   0 ALERT type 5 Informative message, check
```

It is advisable to attempt to resolve as many as possible of the alerts in all categories. Often the minor alerts point to easily fixed oversights, errors and omissions in your CIF or refinement strategy, so attention to these fine details can be worthwhile. In order to resolve some of the more serious problems it may be necessary to carry out additional measurements or structure refinements. However, the purpose of your study may justify the reported deviations and the more serious of these should normally be commented upon in the discussion or experimental section of a paper or in the "special_details" fields of the CIF. checkCIF was carefully designed to identify outliers and unusual parameters, but every test has its limitations and alerts that are not important in a particular case may appear. Conversely, the absence of alerts does not guarantee there are no aspects of the results needing attention. It is up to the individual to critically assess their own results and, if necessary, seek expert advice.

Publication of your CIF in IUCr journals

A basic structural check has been run on your CIF. These basic checks will be run on all CIFs submitted for publication in IUCr journals (*Acta Crystallographica*, *Journal of Applied Crystallography*, *Journal of Synchrotron Radiation*); however, if you intend to submit to *Acta Crystallographica Section C* or *E*, you should make sure that full publication checks are run on the final version of your CIF prior to submission.

Publication of your CIF in other journals

Please refer to the *Notes for Authors* of the relevant journal for any special instructions relating to CIF submission.

PLATON version of 24/07/2014; check.def file version of 24/07/2014

Datablock mo_ph15_0m - ellipsoid plot

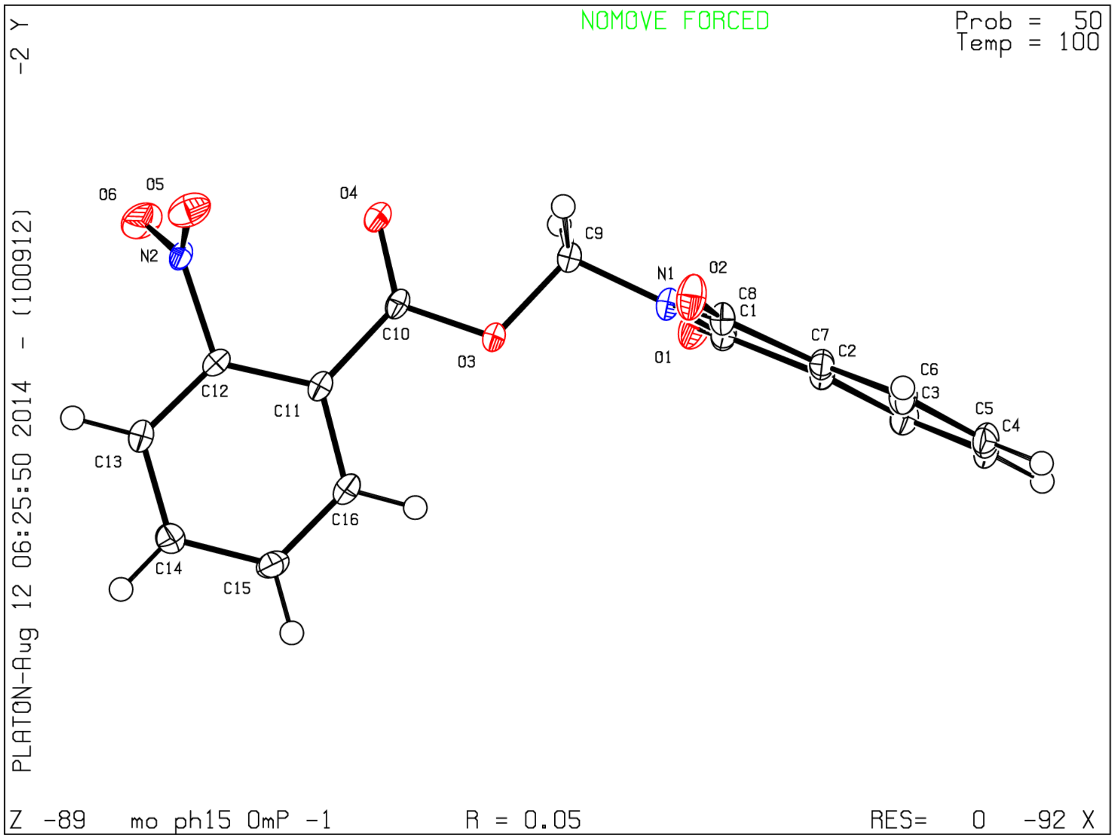

checkCIF/PLATON report

Structure factors have been supplied for datablock(s) mo_ph17a_0m

THIS REPORT IS FOR GUIDANCE ONLY. IF USED AS PART OF A REVIEW PROCEDURE FOR PUBLICATION, IT SHOULD NOT REPLACE THE EXPERTISE OF AN EXPERIENCED CRYSTALLOGRAPHIC REFEREE.

No syntax errors found. CIF dictionary Interpreting this report

Datablock: mo_ph17a_0m

```
Bond precision:  C-C = 0.0035 A         Wavelength=0.71073

Cell:            a=13.2981(19)      b=7.5485(11)      c=29.095(3)
                 alpha=90           beta=90           gamma=90
Temperature:     297 K

                Calculated                Reported
Volume          2920.6(7)                 2920.6(7)
Space group     P b c n                   P b c n
Hall group      -P 2n 2ab                 -P 2n 2ab
Moiety formula  C16 H10 N2 O6             C16 H10 N2 O6
Sum formula     C16 H10 N2 O6             C16 H10 N2 O6
Mr              326.26                    326.26
Dx,g cm-3       1.484                     1.484
Z               8                         8
Mu (mm-1)       0.116                     0.116
F000            1344.0                    1344.0
F000'           1344.81
h,k,lmax        18,10,41                  18,10,40
Nref            4306                      4242
Tmin,Tmax       0.988,0.991               0.929,0.991
Tmin'           0.929

Correction method= MULTI-SCAN

Data completeness= 0.985          Theta(max)= 30.100

R(reflections)= 0.0570( 1707)     wR2(reflections)= 0.2126( 4242)

S = 0.928                  Npar= 217
```

The following ALERTS were generated. Each ALERT has the format
test-name_ALERT_alert-type_alert-level.
Click on the hyperlinks for more details of the test.

Alert level C

```
PLAT026_ALERT_3_C Ratio Observed / Unique Reflections too Low ....         40 %
PLAT230_ALERT_2_C Hirshfeld Test Diff for    N2     --  C14     ..        5.5 su
PLAT242_ALERT_2_C Low       Ueq as Compared to Neighbors for .....         N2 Check
PLAT906_ALERT_3_C Large K value in the Analysis of Variance ......     17.632 Check
PLAT906_ALERT_3_C Large K value in the Analysis of Variance ......      2.377 Check
PLAT911_ALERT_3_C Missing # FCF Refl Between THmin & STh/L=  0.600          6 Report
```

Alert level G

```
PLAT063_ALERT_4_G Crystal Size Likely too Large for Beam Size ....       0.64 mm
PLAT072_ALERT_2_G SHELXL First  Parameter in WGHT Unusually Large.       0.11 Report
PLAT910_ALERT_3_G Missing # of FCF Reflections Below Th(Min) .....          1 Report
PLAT912_ALERT_4_G Missing # of FCF Reflections Above STh/L=  0.600         56 Note
```

---

0 **ALERT level A** = Most likely a serious problem - resolve or explain
0 **ALERT level B** = A potentially serious problem, consider carefully
6 **ALERT level C** = Check. Ensure it is not caused by an omission or oversight
4 **ALERT level G** = General information/check it is not something unexpected

0 ALERT type 1 CIF construction/syntax error, inconsistent or missing data
3 ALERT type 2 Indicator that the structure model may be wrong or deficient
5 ALERT type 3 Indicator that the structure quality may be low
2 ALERT type 4 Improvement, methodology, query or suggestion
0 ALERT type 5 Informative message, check

It is advisable to attempt to resolve as many as possible of the alerts in all categories. Often the minor alerts point to easily fixed oversights, errors and omissions in your CIF or refinement strategy, so attention to these fine details can be worthwhile. In order to resolve some of the more serious problems it may be necessary to carry out additional measurements or structure refinements. However, the purpose of your study may justify the reported deviations and the more serious of these should normally be commented upon in the discussion or experimental section of a paper or in the "special_details" fields of the CIF. checkCIF was carefully designed to identify outliers and unusual parameters, but every test has its limitations and alerts that are not important in a particular case may appear. Conversely, the absence of alerts does not guarantee there are no aspects of the results needing attention. It is up to the individual to critically assess their own results and, if necessary, seek expert advice.

Publication of your CIF in IUCr journals

A basic structural check has been run on your CIF. These basic checks will be run on all CIFs submitted for publication in IUCr journals (*Acta Crystallographica*, *Journal of Applied Crystallography*, *Journal of Synchrotron Radiation*); however, if you intend to submit to *Acta Crystallographica Section C* or *E*, you should make sure that full publication checks are run on the final version of your CIF prior to submission.

Publication of your CIF in other journals

Please refer to the *Notes for Authors* of the relevant journal for any special instructions relating to CIF submission.

PLATON version of 24/07/2014; check.def file version of 24/07/2014

Datablock mo_ph17a_0m - ellipsoid plot

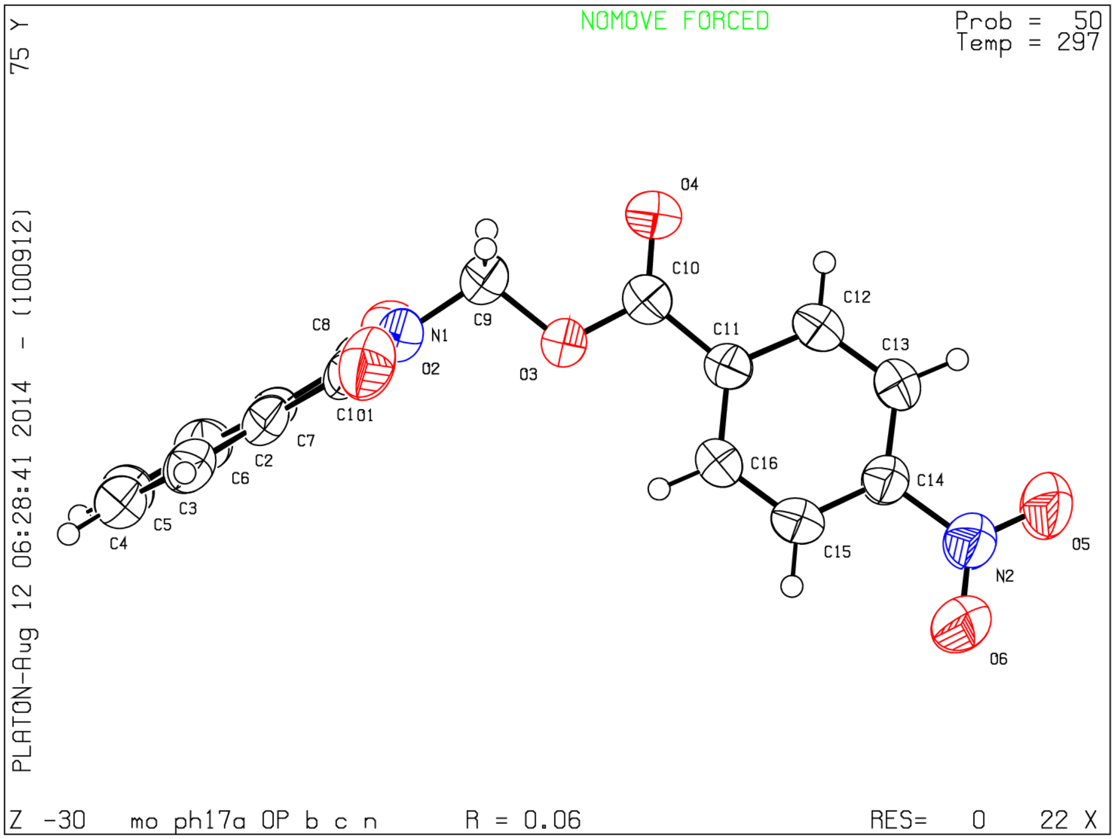

Supplement: S1 Supporting Information — (PDF) [file pone.0119440.s001.pdf]
